# Human-Driven Microbiological Contamination of Benthic and Hyporheic Sediments of an Intermittent Peri-Urban River Assessed from MST and 16S rRNA Genetic Structure Analyses

**DOI:** 10.3389/fmicb.2017.00019

**Published:** 2017-01-24

**Authors:** Romain Marti, Sébastien Ribun, Jean-Baptiste Aubin, Céline Colinon, Stéphanie Petit, Laurence Marjolet, Michèle Gourmelon, Laurent Schmitt, Pascal Breil, Marylise Cottet, Benoit Cournoyer

**Affiliations:** ^1^Research Group on “Bacterial Opportunistic Pathogens and Environment”, UMR CNRS5557, INRA1418 Ecologie Microbienne, Université Lyon 1, VetAgro SupMarcy L'Etoile, France; ^2^DEEP, INSA LyonVilleurbanne, France; ^3^Institut Français de Recherche pour l'Exploitation de la Mer (IFREMER), SG2M-Laboratoire Santé Environnement et Microbiologie, RBE DépartementPlouzané, France; ^4^LIVE 7362 Centre National de la Recherche Scientifique-ENGEES, LTER – “Zone Atelier Environnementale Urbaine”Strasbourg, France; ^5^Institut National de Recherche en Sciences et Technologies pour l'Environnement et l'Agriculture (IRSTEA), UR HHLYVilleurbanne, France; ^6^UMR5600 “Environnement Ville Société,” École Normale Supérieure de Lyon (ENS) Lyon – DescartesLyon, France

**Keywords:** peri-urban river, benthic and hyporheic sediments, microbial community, high throughput sequencing (HTS), fecal contamination

## Abstract

Rivers are often challenged by fecal contaminations. The barrier effect of sediments against fecal bacteria was investigated through the use of a microbial source tracking (MST) toolbox, and by Next Generation Sequencing (NGS) of V5-V6 16S rRNA gene (*rrs*) sequences. Non-metric multi-dimensional scaling analysis of V5-V6 16S rRNA gene sequences differentiated bacteriomes according to their compartment of origin i.e., surface water against benthic and hyporheic sediments. Classification of these reads showed the most prevalent operating taxonomic units (OTU) to be allocated to *Flavobacterium* and *Aquabacterium*. Relative numbers of *Gaiella, Haliangium*, and *Thermoleophilum* OTU matched the observed differentiation of bacteriomes according to river compartments. OTU patterns were found impacted by combined sewer overflows (CSO) through an observed increase in diversity from the sewer to the hyporheic sediments. These changes appeared driven by direct transfers of bacterial contaminants from wastewaters but also by organic inputs favoring previously undetectable bacterial groups among sediments. These NGS datasets appeared more sensitive at tracking community changes than MST markers. The human-specific MST marker HF183 was strictly detected among CSO-impacted surface waters and not river bed sediments. The ruminant-specific DNA marker was more broadly distributed but intense bovine pollution was required to detect transfers from surface water to benthic and hyporheic sediments. Some OTU showed distribution patterns in line with these MST datasets such as those allocated to the *Aeromonas, Acinetobacter*, and *Pseudomonas*. Fecal indicators (*Escherichia coli* and total thermotolerant coliforms) were detected all over the river course but their concentrations were not correlated with MST ones. Overall, MST and NGS datasets suggested a poor colonization of river sediments by bovine and sewer bacterial contaminants. No environmental outbreak of these bacterial contaminants was detected.

## Introduction

Ecological quality of aquatic systems can be significantly impaired by fecal pollutions. These can originate from point i.e., wastewater treatment plant (WWTP), slaughterhouse, and non-point sources i.e., crop field runoff, wild animals, leaking septic tank (Field and Samadpour, 2007). They can harbor chemical and microbial contaminants such as parasites (*Giardia intestinalis, Cryptosporidium parvum*), bacteria (*Salmonella enterica, Campylobacter coli* or *C. jejuni*, enterohemorrhagic *Escherichia coli*) and viruses (Ramírez-Castillo et al., 2015). It is thus a priority of several agencies to detect fecal pollution in order to prevent exposure to their potential infectious agents but also propose changes in the activities leading to their releases (USEPA, 2005). For a century, fecal indicators (FI) such as *E. coli* and enterococci have been used in these monitorings (Dufour, 1984; Ashbolt et al., 2001). However, they are poorly effective at differentiating the origin of a fecal pollution (Field and Samadpour, 2007). Furthermore, discrepancies between FI and pathogen distributions have been reported in several studies (Harwood et al., 2005; Wilkes et al., 2011; Jokinen et al., 2012).

During the last decade, several methods have thus been developed to improve the reliability of FI. A “Microbial Source Tracking (MST)” of bacterial taxa, which can be indicative of the presence of certain fecal emitters, has been developed. One of the most promising MST targets appeared to be the 16S rRNA (*rrs*) gene sequences from *Bacteroidales* (Roslev and Bukh, 2011). *Bacteroidales* are numerous in the intestinal tract of mammalians and several species are host-specific. They are often anaerobic and thus not likely to grow in most outdoor situations (Fiksdal et al., 1985; Bernhard and Field, 2000a; Fogarty and Voytek, 2005). Several *Bacteroidales* markers have been designed to detect fecal pollution from human (Seurinck et al., 2005), ruminant (Reischer et al., 2006), pig (Mieszkin et al., 2009), and wild animals (Fremaux et al., 2010; Marti et al., 2011a, 2013b). Correlations with the occurrence of human pathogens have been observed (Fremaux et al., 2009; Staley et al., 2012; Marti et al., 2013a; Wilkes et al., 2013, 2014). Ruminant-specific MST markers BacR and CF128, designed by Reischer et al. (2006) and Bernhard and Field (2000b), respectively, and the human-specific marker HF183, were found positively correlated to an occurrence of *Salmonella* cells (Fremaux et al., 2009; Marti et al., 2013a; Wilkes et al., 2014). However, despite these cases, as for FI, no strong correlation has been highlighted between MST markers and most pathogens. The main reasons are likely (1) differences in the tropism of MST targets and pathogens for outdoor habitats and (2) a lack of sensitivity of the real time PCR method leading to false negative results (Marti et al., 2013a).

Only a few studies have investigated the bacterial quality of benthic and hyporheic sediments of rivers in a MST scheme (Frey et al., 2015; Bradshaw et al., 2016). Benthic sediment refers to the first cm of the river bed, and is characterized by the presence of photosynthetic organisms. Below the benthic sediment, waters can infiltrate at variable flow and lead to a transfer, amongst others, of organic matter, biological agents, and oxygen. These transfers can occur at variable depth according to the nature of the river bed including the presence of sand, clay and gravel or rocks (Likens, 2010). This zone of transfer is named the hyporheic zone. The benthic and hyporheic zones harbor most of the biomass of a river including microbial biofilms (Fischer and Pusch, 2001). River biofilms are involved in key activities such as the degradation of organic matter, and can contribute at 76–96% of the total biological activities (Vaque et al., 1992; Naegeli and Uehlinger, 1997; Craft et al., 2002; Seitzinger et al., 2006). Benthic and hyporheic sediments act as filters concentrating nutrients, pollutants, and trapping particles including micro-organisms. This filter effect will depend upon the nature of the river bed media including size of its physical components. These will affect the porosity of the bed and impact water flow in the hyporheic zone (Gibert et al., 1995; Maazouzi et al., 2013). Disturbance of these zones can lead to a decrease of the ecological quality of a river (Lafont et al., 2009).

Artificial infiltration columns of sediments were designed to infer the decay or establishment of fecal bacteria in river sediments. These investigations showed the decay of fecal bacteria to be related to factors such as predation, salinity and UV (Bradford et al., 2013). However, an increasing of organic-C content of sediments was found correlated with a significant development of fecal bacteria (Bradford et al., 2013). However, in natural systems, endogenous biofilms and natural clogging could prevent such developments (Battin and Sengschmitt, 1999). In fact, bacterial contaminants coming from human or animal hosts are not expected to adapt to river ecosystems because of their growth requirements such as a warm temperature and particular nutrients and oxygen concentrations. Fecal bacteria should thus be mainly recovered transiently from surface waters and benthic sediments, and rarely get established in the hyporheic zone. Accordingly, few bacterial groups observed among sewers and animal wastes contaminating rivers are expected to be recovered from sediments. Nevertheless, some studies indicated that river bed sediments could be significant reservoirs of certain bacterial pathogens. These could get re-suspended when the river flow reaches a critical level (Brookes et al., 2004; Jung et al., 2014; Frey et al., 2015). These reservoirs can thus be significant in the global epidemiology of waterborne pathogens.

In this study, transfers of FI and other allochtonous bacteria from agricultural and urban sites into a small peri-urban stream, named Chaudanne, and being part of the Yzeron watershed, were investigated. MST and 16S rRNA genetic structure analyses of bacteriomes were used to monitor these transfers. Benthic and hyporheic sediments, and surface waters from three segments of the river were analyzed: (1) one being close to its source, (2), one highly impaired by agricultural and urban activities, and (3) one located at the outlet of the Yzeron River. These analyses revealed strong differentiations of bacteriome genetic structures between surface waters, and the benthic and hyporheic sediments. Bacterial contaminants coming from wastewaters and animals, monitored by NGS and MST analyses, did not show massive transfers into sediments that would drastically change the bacteriomes. Nevertheless, sediments impacted by urban activities showed bacterial genera known to harbor potential human pathogens. These taxa appeared to be in low numbers, and not likely to outgrow the indigenous bacteria. The *rrs* NGS datasets revealed novel bacterial operating taxonomic units (OTU) that could be used in the design of MST markers.

## Materials and methods

### Experimental site and samplings

**Figure 1:**
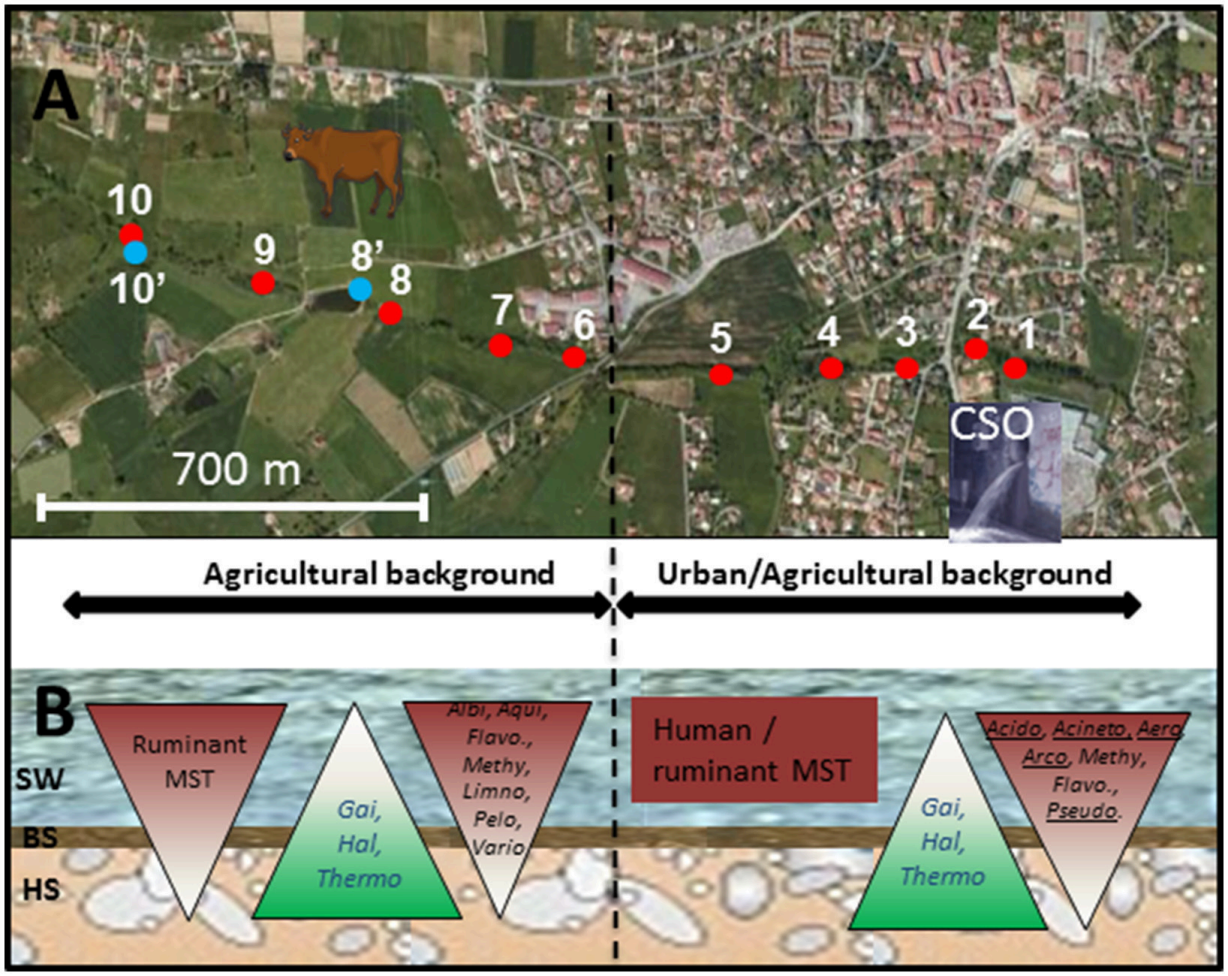
**Map showing the sites sampled along the Chaudanne river**. Sites are further detailed in Table 1. **(A)** Analyzed segment of the Chaudanne River; Yzeron river outlet is about 20 km downstream of point 1. **(B)** Scheme of the Chaudanne river section showing the different compartments and the vertical distribution of ruminant and human specific Bacteroidales MST markers and genera inferred from NGS 16S rRNA gene analyses. SW, Surface Water; BS, Benthic Sediment; HS, Hyporheic Sediment. Acido, *Acidobacteria*; Aero, *Aeromonas*; Acineto, *Acinetobacter*; Albi, *Albidiferax*; Arco, *Arcobacter*; Flavo, *Flavobacterium*; Gai, *Gaiella*; Hal, *Haliangium*; Thermo, *Thermoleophilum*; Pelo, *Pelomonas*; Pseudo, *Pseudomonas*; Vario, *Variovorax*. Underlined genera showed higher relative counts in wastewater than in surface water.

The Yzeron watershed was investigated in this study through analyses of its tributary named La Chaudanne and of its outlet being connected to the Rhône River (Namour et al., 2015). Surface of the Yzeron watershed is of 129 km^2^. The distance between the La Chaudanne tributary and the Yzeron outlet (a concrete run) is about 20 km-long. The Chaudanne intermittent stream culminates at 443 m and is located at Grezieu-La-Varenne (Western Lyon). It is 4.4 km-long (slope of 0.05 m/m). A map of the section of the Chaudanne river area that was investigated is shown in Figure 1. This river is impacted by combined sewer overflow (CSO) devices. Averaged flow values of the river and overflow volumes of the Chaudanne CSO are indicated in Table S1. The twelve river sites (1–10 plus sites 8′ and 10′) were sampled at winter time (averaged water temperature of 5°C, pH of 7.2, and electrical conductivity of 0.308 μS/m), physico-chemical parameters are summarized in Table S2. Surface waters, benthic, and hyporheic sediments, were sampled from the source of the Chaudanne stream toward an area highly impaired by combined sewer and stormwater overflows (located 3 km downstream its source) (Table 1 and Table S2). Hydrological indices defining the impact of CSO events on the river showed significant differences between the low flow (LFS) and high flow (HFS) seasons. The dilution ratio of CSO waters over the natural flow was around 0.22 in HFS and of 3.4 during LFS. Samplings were performed during HFS. Site 10 is the source of the Chaudanne River; sites 9 through 6 are located in agricultural areas with reared animals and crops; sites 5 to 1 are peri-urban with both agricultural and urban activities (Figure 1). The outlet of the watershed was sampled at 500 m before joining the Saone River. Samples were collected in the middle of run-riffle geomorphic units. Benthic sediments were recovered over a 20 cm large transect going from one side of the river bed (about 1 m large) to the other side. Additionally, a wastewater sample was taken from the sewer connected to the CSO localized between sampling points 2 and 3 (Table 1). A Bou-Rouch pump attached to a perforated zinced iron pipe was used to retrieve hyporheic sediments at a depth of −30 cm from the top sediment layer. Benthic sediments (0 to −5 cm) were directly transferred into a bucket. Sediments were put at 105°C for 48 h, and their dry weights were then measured. Some physico-chemical parameters (pH, temperature, electrical conductivity) were measured directly on the surface and hyporheic waters. All samples were held at 4°C and analyzed within 24 h of sample collection.

**Table 1:** **Sampling sites along the Chaudanne River**.

**Site**	**Characteristics (category)**
1	150 m downstream the main combined sewer overflow (CSO) of Grezieu-la-Varenne; close to houses and a commercial mall (rural-urban)
2	20 m downstream the CSO; close to a pedestrian path and a busy road (rural-urban)
3	150 m upstream the CSO; along a pasture field with a horse (rural-urban)
4	225 m upstream the CSO; close to houses, corn and pasture fields; presence of donkeys (rural-urban)
5	390 m upstream the CSO; close to a road; downstream a stormwater overflow and a sewer overflow connected to a residential lift pump system; at the end of a corn field (rural-urban)
6	625 m upstream the CSO; 30 m upstream site 5; at the end of residential gardens, and a pasture field (few meters); about 100 m downstream a factory (rural)
7	775 m upstream the CSO; along a pasture field and upstream the factory of site 6 (rural)
8	1000 m upstream the CSO; downstream a detention pond pouring into the Chaudanne River, along a corn field (rural)
8'	Surface water of the detention pond of site 8 (rural)
9	upstream detention pond pouring into La Chaudanne, middle of a cattle herd
10	1400 m upstream the CSO; closest site to the source; upstream the cattle herd of site 9 and at the end of a pasture field, (most pristine site; source)
10'	Brook going through the pasture field of site 10; outlet connected to the Chaudanne River; sampling point at about 50 m from site 10 (rural)

Bacterial platings were performed on serially diluted (0.08% NaCl) surface waters and sediments. Total heterotroph numbers were estimated on 1/10 diluted TSA (tryptic soy agar). *E. coli* and thermotolerant fecal coliform numbers were estimated by plating on Rose Gal-BCIG medium (Biokar) and incubation at 42°C for 24 h. Blue/indigo-forming colonies were considered as *E. coli* and pink-forming colonies as the other fecal coliforms. Bacterial concentrations were expressed in CFU/100 mL or/g of dry weight sediment. All these analyses were performed in triplicate.

### DNA extractions and MST quantitative PCR

DNA were extracted from filtered waters or sediments using the FastDNA SPIN Kit for Soil (MP Biomedicals, Illkirsch, France) according the manufacturer's instructions, with some modifications specified below. About 600–700 mg (wet weight) of benthic and hyporheic sediments were used per DNA extraction. For water samples, prior to DNA extraction, about 100–300 mL were filtered through 0.45 μm cellulose acetate membrane and stored at −20°C. Filters were placed in lysing matrix tubes, frozen in liquid nitrogen and ground with a sterile tip. Finally, filled lysing matrix tubes were processed at 4°C in a TissueLyzer II (Qiagen) for 75 s at maximum speed. Then, DNA was extracted as for sediment samples with the FastDNA SPIN Kit. One additional wash with SEW-S M solution was done and final elution of DNA was performed twice with 50 μL DES solution. These DNAs were quantified using a spectrophotometer with absorbance readings performed at 260 and 280 nm or by using the Picogreen quantification procedure (BioRad). MST qPCR amplifications of general and host-specific *Bacteroidales* markers were performed as described in Mieszkin et al. (2009; 2010; Table S3) using a Stratagene Mix 3000P PCR cycler (Stratagene). MST markers used were detected using Taqman chemistry for Rum-2-Bac (ruminant specific), Pig-2-Bac (pig specific), and total Bacteroidales and SYBR Green chemistry for the HF183 marker (human specific). Analyses have been done on normalized DNA concentrations set at 5 ng. A standard curve was generated using serial 10-fold dilutions (ranging from 1.6 × 10^7^–1.6 × 10^0^ gene copies per real-time PCR, with a quantification limit of 1.6 target copies per reaction) of a plasmid preparation containing the targeted sequence of *Bacteroidales* (partial 16S rRNA gene sequence insert). Plasmid DNA was extracted from overnight cultures of *E. coli* using the QIAprep® Miniprep kit (Qiagen), linearized with the *Not*I enzyme (Fermentas) and quantified as described in the above section.

The presence/absence of PCR inhibitors in the DNA extracts was verified using an Internal Positive Control (IPC) (Applied Biosystem, France). DNA samples (2 ng) were tested with the IPC at no-dilution, 1/10 and 1/100 dilution. The lowest dilution of DNA without inhibition was used in the PCR reactions. For each run, negative controls (no template DNA) were performed. All samples were tested in duplicate on separate plates. Data analysis was performed using the Mx Pro Software (Stratagene). Specificity of PCR products with HF183 was checked by melting-curve analysis (Tm of 75.5°C). MIQE guidelines were used in the quantification assessments (Bustin et al., 2009) and reactions showing less than 3 copies were considered below the limit of detection (LOD). MST qPCR data sets were expressed as the mean of copy number of DNA targets/100 mL of water or g of sediment. When needed, data were normalized with the total *Bacteroidales* MST counts. Analyses were performed in triplicate.

### NGS of 16S rRNA gene PCR products

DNA extracts from surface waters and benthic and hyporheic sediments collected at the source of the Chaudanne river (site 10), from the area impaired by combined sewer and stormwater overflows (sites 1 and 2), and from the Yzeron outlet were used in these analyses. The V5 and V6 segments of 16S rRNA genes (*rrs*) were PCR amplified from these extracts using forward 5′-AGGATTAGATACCCTGGTA-3′ and reverse 5′-CAACACGAGCTGACGAC-3′ primers, and sequenced by DNA Vision (Gosselies, Belgium) according to De Filippo et al. (2010). Pyrosequencing was carried out on a 454 Life Sciences Genome Sequencer FLX instrument (Roche) following Titanium chemistry. The generated sequences were filtered with the Mothur package in order to remove chimeric sequences, primers, barcodes, and limit the dataset to sequences of a minimum length of 200 bp (average length = 260 bp) (Schloss et al., 2009). Singletons were kept in the datasets. The SILVA v119 16S Bacterial reference library was used for taxonomic allocation of the OTUs (https://www.arb-silva.de/documentation/release-119/). Cut off was set at 0.01 (99% identity) for OTU classification. OTU sequences have been deposited in GenBank under the accession numbers KU258887 to KU284840. Alpha diversity indices (Shannon and Chao) and beta diversity (Bray-Curtis) were computed using the Mothur package. Contingency table encompassing OTU affiliations was imported into the Explicet software (Robertson et al., 2013) in order to generate a heatmap illustrating genus distribution patterns between river compartments. DNA sequences were further compared to an additional database built by importing sequences from GenBank. This database included 16S rRNA gene sequences from waterborne pathogens listed by the World Health Organization and French Agency for Food, Environmental and Occupational Health & Safety (ANSES) (WHO, 2003; Lagriffoul et al., 2009). The 16S rRNA gene accession numbers of these pathogens are indicated in Table S4. Only GenBank sequences from isolates with a well-established classification were considered. Alignments were performed using the Blast+ application (Camacho et al., 2010). Default settings were used except the *e*-value which was set at 1e^−30^. Mega software (version 6.06) was used to construct a *Bacteroides* phylogenetic tree including the MST target sequences. Blast searches making use of the qPCR primer sequences targeting HF183 and Rum-2-Bac (*Bacteroides*) and Pig-2-Bac (*Prevotella*) were performed, and allowed extracting their matching full length *rrs* sequences [accession number AJ408983 (HF183); JX096090 (Rum-2-Bac) and AF371872 (Pig-2-Bac)] for this phylogenetic analysis. One Thousand Bootstrap replicates were done to test the reliability of phylogenetic tree branches (Tamura et al., 2013). This tree was completed using a set of representative *Bacteroides* related OTU sequences extracted from the V5-V6 *rrs* NGS database of this work.

### Statistical tests

ANOVA, Kolmogorov-Smirnov, Kruskal-Wallis, Fisher's exact tests, and Spearman correlation tests were done using the R software version 3.1.3 (R Core Team, 2015). Cluster and non-metric multi-dimensional scaling (NMDS) analyses were performed using R with the Vegan Package (Oksanen et al., 2015).

## Results

### Physico-chemical and fecal bacterial indicators

Physico-chemical values are summarized in Table S2. These analyses revealed no significant difference between the three sampling areas (i.e., pristine, mainly agricultural, mixed agricultural/urban backgrounds) using ANOVA (data not shown). For pH, values ranged from 6.8 to 7.6 in surface water, 6.7 to 7.4 for the benthic and 6.5 to 7.5 for the hyporheic waters (Table S1). A significant difference was observed for the electrical conductivity between benthic and hyporheic waters (*p* < 0.05). Conductivity values ranged from 0.257 to 0.324 mS / m (average 0.297 ± 0.027 mS / m) for surface water samples, 0.184 to 0.310 mS / m for benthic (average 0.262 ± 0.046 mS / m) and 0.236 to 0.60. 1 mS / m for hyporheic waters (average 0.368 ± 0.127 mS / m). These differences between the benthic and hyporheic waters are in agreement with those reported in Namour et al. (2015). It is to be noted that these differences were previously found inversely related to the concentrations of dissolved oxygen. Namour et al. (2015) reported about 3.7 mg dissolved oxygen per L in the hyporheic zone of one of the segment of the Chaudanne River used in this study, while it was of about 8.5 mg for the benthic waters. This is likely related to a significant consumption of oxygen by hyporheic inhabitants including bacteria. Darcy flows indicated reduced transit time of waters in the hyporheic zone of this river that can favor the consumption of organic-C and other chemicals (Namour et al., 2015).

**Figure 2:**
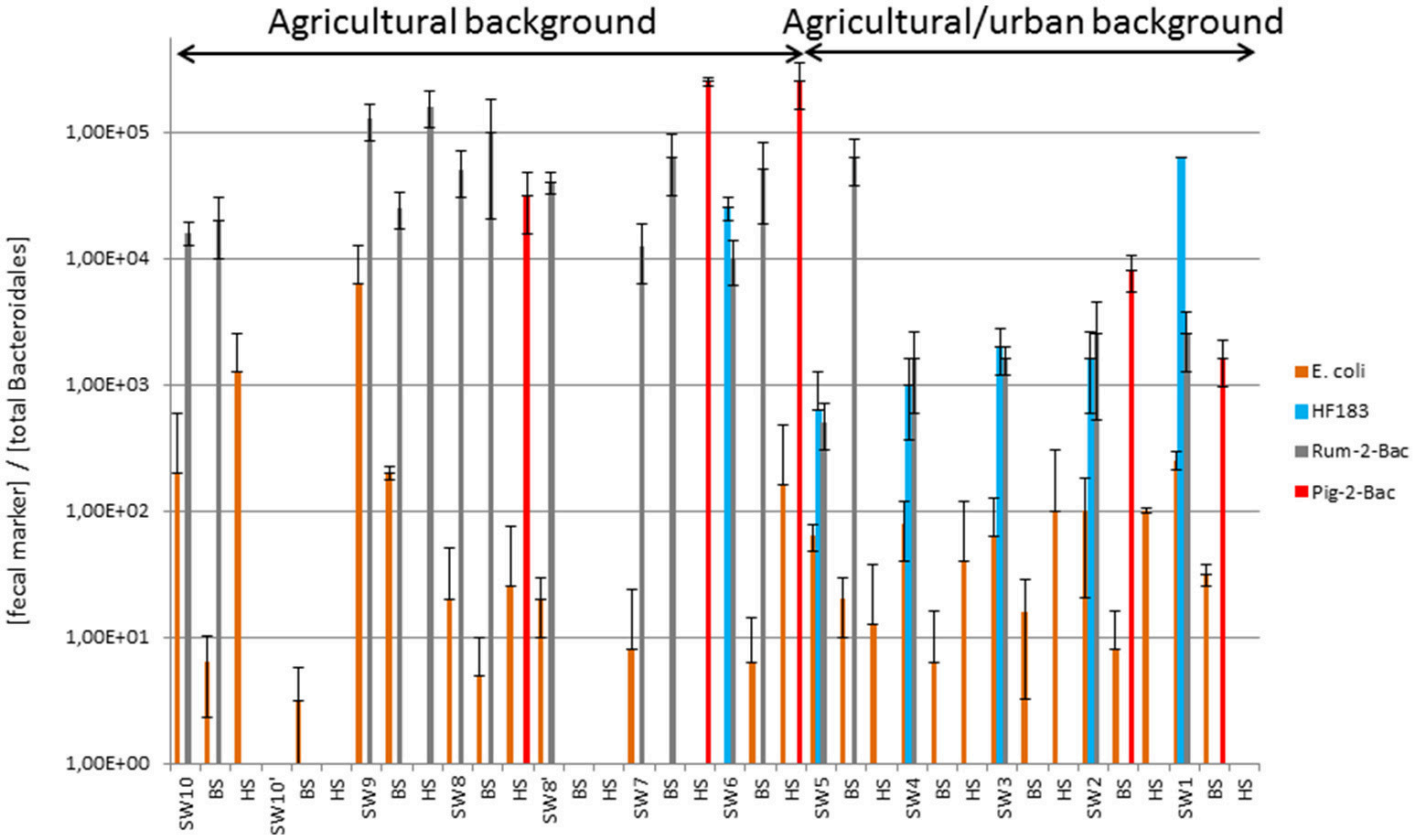
**Relative counts of fecal bacteria and MST host-markers expressed over total Bacteroidales concentrations measured along the Chaudanne River watercourse and bed sediments**. See Table 1 for a description of the sampling sites. Datasets used for the computations are shown in Table S5. SW, surface water, BS, benthic, and HS, hyporheic sediments.

Bacterial counts for the river samples are summarized in Table S5. Total thermotolerant coliforms (TTC) as well as *E. coli* have been detected at all sampling sites (Table S5, Figure 2). However, they were not detected among all compartments of all sites (surface waters, benthic, and hyporheic sediments). For the pristine site (named 10), TTC and *E. coli* were found in all compartments with concentrations ranging from 1.1 to 1.5 Log_10_ units CFU/100 mL or g. For the set of samples belonging to the agricultural background (i.e., sites 6 to 9, 8′, and 10′), TTC and *E. coli* were also found at least one time in each compartment but they were not necessarily detected in the 3 compartments of each site. When detected, TTC and *E. coli* concentrations ranged, respectively, from 1.5 to 2.6 and 1.5 to 2.5 Log_10_ CFU/100 mL in surface water 0.8 to 2.2 and 0.8 to 2.4 Log_10_ CFU/g of benthic sediment, and 0.8 to 1.2 Log_10_ CFU/g of hyporheic sediment. For the mixed agricultural/urban background, all sites were positive for TTC and *E. coli*. For all of them, the FI were found in all compartments except the hyporheic sediment of site 1 where *E. coli* was not detected. When detected, concentrations of TTC and *E. coli* ranged, respectively, from 2.1 to 2.9 and 2.1 to 2.8 Log_10_ CFU/100 mL in surface water, 1.6 to 2.6 and 0.8 to 2.3 Log_10_ CFU/g of benthic sediment, and 0.5 to 2.9 and 0.5 to 2.8 Log_10_ CFU/g of hyporheic sediment. These TTC and *E. coli* counts were normalized per number of total Bacteroidales DNA targets (Figure 2). This allowed Kruskal-Wallis statistical comparisons of TTC and *E. coli* relative counts between water and sediments. These analyses revealed no significant differences in TTC and *E. coli* numbers between surface water and hyporheic sediment when looking at all samples from the watercourse. However, analyses restricted to the agricultural/urban background showed significant differences in the *E. coli* relative counts (*p* = 0.04) with the highest values obtained for the surface waters. The TTC relative counts were found significantly higher in the agricultural/urban background (i.e., sites 1–5) than in the agricultural one (i.e., sites 6–9) (*p* = 0.02). No significant difference was observed between compartments for both *E. coli* and TTC except when we focused on mixed agriculture/urban area where *E. coli* showed higher ratio in surface water than in benthic sediment (*p* = 0.04). At the Yzeron River outlet (concrete run with openings on the underground compartment), surface water showed 4.1 Log_10_ CFU/100 mL of TTC, and 2.4 Log_10_ CFU of TTC/g of sediment were obtained for benthic sediment. The hyporheic sediment had a TTC count of 1.6 Log_10_ CFU/g of sediment. For *E. coli*, concentrations for surface water, benthic, and hyporheic sediment were 3.6 Log_10_ CFU/100 mL, 1.4 Log_10_ CFU/g and below limit of detection, respectively.

### MST of river contaminants

Figures 1, 2 summarize the main trends observed in the MST datasets presented in Table S5. At the source of the river, the ruminant MST markers were detected in surface water and in benthic sediment but were not detected in hyporheic sediment. Human and pig MST markers were not detected in these “source” samples. For the agricultural background area, all sampling sites were at least positive for one MST marker except site 10′. However, none of them were positive for the three markers and only two sites (6 and 9) were positive for two MST markers. The human MST marker was only detected among surface water of the “agricultural” site 6 (Table S5; Figure 1). This site was close to a few houses that apparently led to significant human contaminations. However, the human MST marker was not detected among benthic and hyporheic sediments of the other agricultural sampling sites (Figure 1). The ruminant MST marker showed the highest prevalence. It was found in 67% of the sampled benthic sediments, and in 17% of the hyporheic ones (site 9). Pig MST marker was also detected in the agricultural background but strictly among sediments and more particularly in the hyporheic ones. Sixty seven percent of the sites were positive for this marker. Among the mixed agricultural/urban backgrounds, human, and ruminant MST markers were found at all sampling sites while the pig marker was found at two sites. The human MST marker was detected only in surface waters. Ruminant MST marker was detected in all sampled surface waters, and in one benthic sediment (site 5, at the border of the cattle area). Pig MST marker was detected only in benthic sediments of sites 1 and 2.

qPCR MST datasets were transformed into binary codes (±) which were then used for testing biases in their distribution patterns according to river compartments and sampling site (Table S6, Figure 1). Human MST marker was more prevalent in surface water (50%) than other compartments (0% for benthic and hyporheic sediments). Ruminant MST marker was also confirmed more prevalent in the sampled surface waters (83%) than the benthic sediments (33%). A significant decrease in the prevalence of ruminant MST marker was observed from the surface waters to the hyporheic zone (8% of positive samples). Pig MST marker showed complex distribution patterns that could not be explained by these statistical tests. Regarding the sampling zone (pristine, agricultural, or urban-agricultural), Fisher's exact tests did not detect any significant differences in the distribution patterns of the MST markers (data not shown). To complete these statistical analyses, Kruskal-Wallis non-parametric tests were performed on the relative counts of MST host-specific markers expressed over the numbers of total *Bacteroidales* cells (Figure 2, Table S5). These relative counts allowed comparisons of MST host-specific markers between surface water and sediments. Relative counts of the human and ruminant specific MST markers were significantly higher among surface water than benthic and hyporheic sediments along the river watercourse (*p* < 0.001 and = 0.01 respectively). No statistical difference could be observed between the relative counts of ruminant specific markers between the benthic and hyporheic sediments (Figure 2). Samples from the agricultural/urban area showed similar distribution patterns for the human (*p* = 0.0013) and ruminant (*p* = 0.016) MST markers, with relative counts being higher in surface water than in sediment. A background effect was also highlighted by the significant differences in the relative counts of human MST markers between all samples of the agricultural and mixed agricultural/urban backgrounds (*p* = 0.03). A significant background effect was also observed when restricting the analysis to the sampled surface waters, for both, the human and ruminant specific MST markers (*p* = 0.02 and 0.04 respectively). Relative counts of ruminant MST marker were significantly higher in the agricultural area than the mixed agricultural/urban one. Pig MST marker showed significantly higher counts among sediments than surface water (*p* = 0.03). Surface water samples had pig MST marker concentrations below the detection limit. Correlations between *E. coli* and TTC plate counts, and MST host-markers were tested by non-parametric Spearman tests. Comparisons between combinations of backgrounds and compartments did not reveal any significant correlations.

### *rrs* bacteriome genetic structure analyses

#### Genetic diversity indices

16S rRNA gene (*rrs*) PCR products were generated from DNA extracts of surface waters and sediments from: (1) the source of the Chaudanne River, (2) the area of the Chaudanne river impaired by both agricultural and urban activities (named Grezieu), and (3) the outlet of the Yzeron River (named YRO). The numbers of high quality DNA reads obtained from surface waters at the source of the river were relatively lower than those from the other samples. This led to the generation of two distinct sets of 16S rRNA gene sequences, in order to increase the resolution of some statistical analyses. The first set harbored a total of 56700 sequences (named the 50K dataset) and considered all the sampling sites. The second set had 99036 sequences (named the 100K dataset; 11040 sequences per sample excluding DNA sequences from the surface water at the source of the river because only 5670 sequences were available for this sample). A sequencing depth of 5000 *rrs* reads per sample was previously shown to capture more than 80% of the trends in richness and evenness (Lundin et al., 2012). Furthermore, 1000 *rrs* reads per sample were found sufficient to resolve more than 90% of Bray–Curtis dissimilarity indices (Savio et al., 2015). OTUs (operational taxonomic unit grouping *rrs* sequences with at least 99% identity, in our study) were defined, and rarefaction curves computed (Figure S1). Both datasets showed OTU saturation curves in line with the origin of the extracted DNA. A good saturation in the number of OTU could be obtained already from the 50K dataset for the surface waters and wastewater *rrs* PCR products. However, much higher OTU diversity was observed among the sediment *rrs* PCR products, and saturation curves were better resolved with the 100K dataset. These observations were in line with the computed diversity indices estimated by the Chao1 (qualitative species richness) and Shannon indices (nonparametric quantitative species richness/evenness) from the 50K dataset (Table S7). Surface waters and wastewater OTU datasets showed a lower Shannon index than BS and HS, independently of the sampling site. Surface water 16S rRNA gene sequences recovered from the outlet of Yzeron River had the lowest OTU Shannon diversity index (value of 3.5). Highest diversity indices (above 5.9) were obtained from the sediment *rrs* datasets, with the Grezieu hyporheic zone showing the greatest diversity (value of 7.5). Surface water from Grezieu also showed the highest richness among all surface water samples with a Shannon index of 5.6. Divergences between the community structures were estimated by the Bray-Curtis index (Table S7). These indices indicated that the surface waters of the Grezieu site were the most impacted by wastewaters taxa while still showing similarities with those at the Yzeron outlet. Most similar samples were those of the benthic sediments recovered from the three areas. Hyporheic sediments at the source of the river and Grezieu showed closer similarities than the ones computed from OTUs recovered from the sediment at the Yzeron outlet (Table S7). This is likely due to the nature of this latter sediment which was collected from an opening among a concrete run.

#### Inferred taxonomic patterns

**Figure 3:**
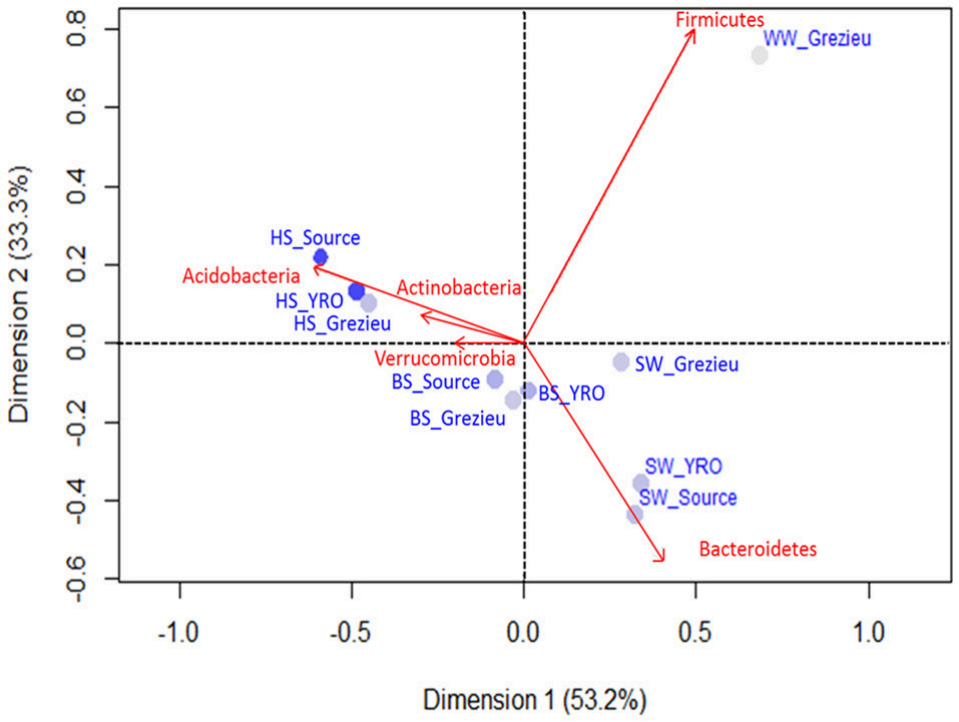
**Correspondence analysis computed from OTU 16S rRNA gene contingency scores at the level of phyla**. The 50K dataset (5670 reads per sample) containing 16S rRNA gene sequences from surface waters (SW), benthic (BS), and hyporheic sediments (HS), and wastewater (WW), sampled along the Chaudanne river, were used (see text). Only the most significant phyla in terms of relative weight are shown (in red). The intensity of the filled circles indicates the absolute contribution of each set of phyla patterns.

The relative occurrences of bacterial phyla inferred from *rrs* sequences between river sites and compartments are summarized in Figure S2. In the 50K dataset, Proteobacteria appeared to be the most prevalent phylum for all sites with percentage ranging from 46.4% of the reads among the Source surface water to 67.6% in YRO benthic sediment, followed by Bacteroidetes, Acidobacteria and Actinobacteria. The wastewater OTU dataset was differentiated from the river samples by a high number of reads allocated to the Firmicutes. This phylum was also detected in the dataset from the Grezieu surface waters (agricultural/urban area) but to a much lesser extent. For all sampling areas, reads of Acidobacteria increased in number from surface water toward the hyporheic zone. These community structures at the phylum scale were compared by a correspondence analysis (Figure 3). Greatest similarities in the distribution of reads between phyla were found among a same compartment (SW, BS, or HS) whatever the sampling site. Furthermore, OTUs of the Acidobacteria and Actinobacteria were found major components of the hyporheic sediments (Figure 3 and Figure S2). Surface waters showed significant numbers of reads that were allocated to the Bacteroidetes. More Bacteroidetes DNA imprints were recovered from surface waters at the source and YRO outlet sites than the Grezieu one. Reads allocated to Firmicutes were associated with the wastewater sample (Figure 3).

**Figure 4:**
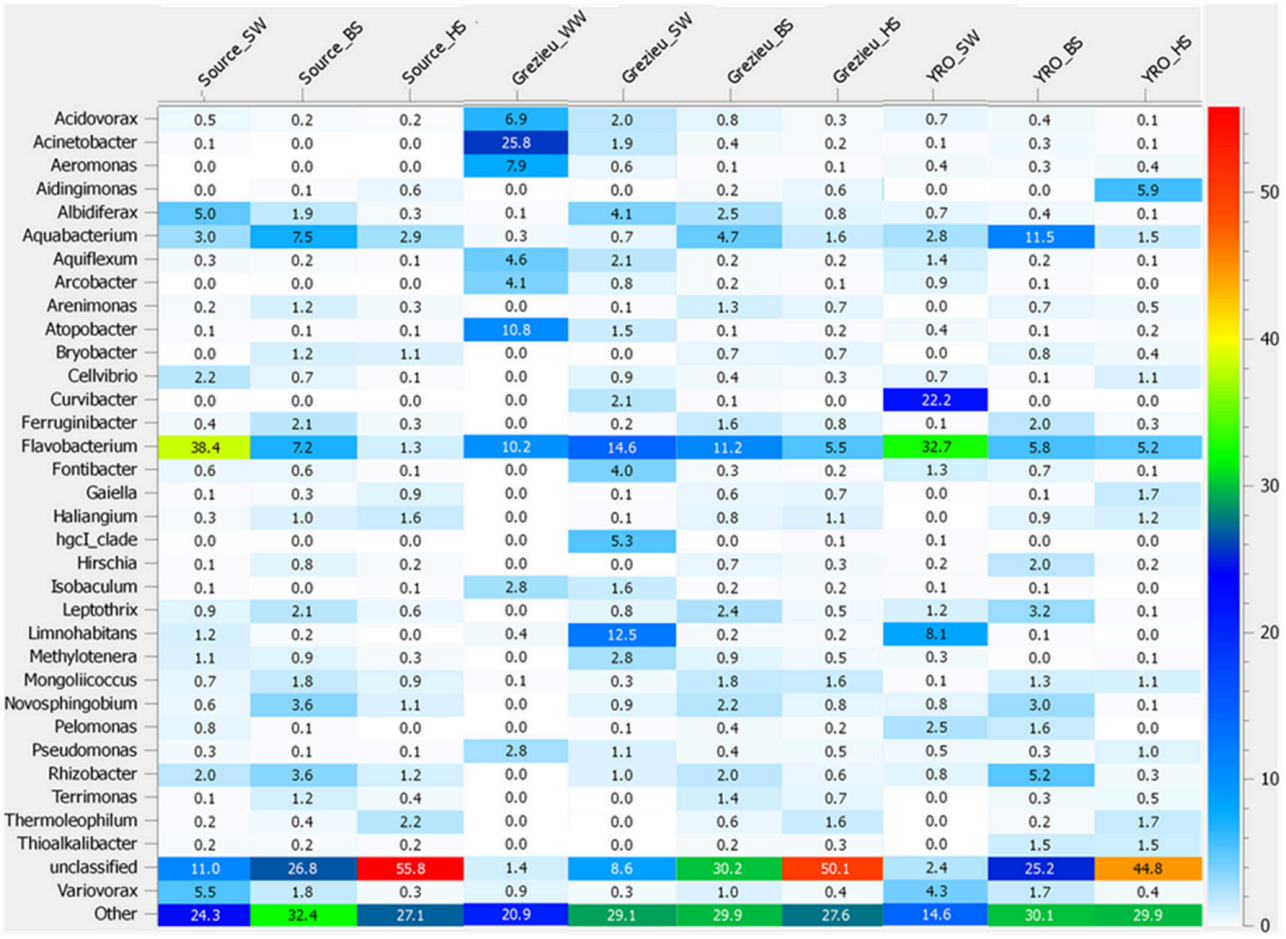
**Heatmap illustrating the significance of some genera inferred from the 16S rRNA gene dataset recovered from river and wastewater samples**. The 50K dataset (5670 reads per sample) containing 16S rRNA gene sequences from surface waters (SW), benthic (BS), and hyporheic sediments (HS), and wastewater (WW), sampled along the Chaudanne river, were used (see text). Genera representing less than 0.4% of the full dataset were merged and grouped into the term “Other.”

Further OTU analyses were performed at the genus level. Distribution patterns of the most prevalent genera according to *rrs* imprints are indicated in Figure 4. The most widely distributed genus was *Flavobacterium* (Bacteroidetes) with an occurrence ranging from 1.3 to 38.4%. The highest numbers of reads were recorded in surface waters from the source and at the outlet of the watershed (YRO site). Reads from *Aquabacterium* (Proteobacteria) were also detected in all samples, and their relative counts ranged from 0.3 to 11.5% with the highest score observed among YRO benthic sediments. Among wastewaters, high numbers of reads were allocated to *Acinetobacter, Atopobacter, Aeromonas*, and *Acidovorax* (Figure 4). Most significant number of *Pseudomonas* (Proteobacteria) *rrs* reads was also recorded in wastewaters (2.8%). Interestingly, a distribution gradient of the reads allocated to the *Pseudomonas* was recorded from the surface water to the river bed sediments at the Grezieu site (0.4–0.5% in sediment and 1.1% in surface water). This gradient was in line with a transfer of wastewater *Pseudomonas* into the surface waters and sediments. *Aeromonas* (Proteobacteria) OTUs also showed similar distribution patterns at the Grezieu site as well as *Acidobacteria* (Acidobacteria), *Acinetobacter* (Proteobacteria), *Arcobacter* (Proteobacteria), *Flavobacterium* (Bacteroidetes), and *Methylotenera* (Proteobacteria) (Figure 1). This confirmed the likely transfer of some wastewater taxa into sediments. Absence of some of these taxa e.g., *Aeromonas* and *Arcobacter* (Figure 4) among the source samples further supported this inference. At the source, several genera also showed a negative distribution gradient from surface water to hyporheic sediment e.g., *Albidiferax* (Proteobacteria), *Aquiflexum* (Bacteroidetes), *Limnohabitans* (Proteobacteria), *Pelomonas* (Proteobacteria), and *Variovorax* (Proteobacteria) (Figures 1, 4), suggesting significant and persistent emissions from the surrounding environment. At the YRO site, most of the genera with a negative distribution gradient among the other sites showed the same trends, with the exception of *Aeromonas, Acinetobacter*, and *Pseudomonas rrs* reads (Figure 4). Interestingly, a positive gradient was recorded from surface water to hyporheic sediment for reads allocated to the genera *Gaiella* (Actinobacteria), *Haliangium* (Proteobacteria), and *Thermoleophilum* (Actinobacteria) for the three sampling sites (Figures 1, 4). Similarly, when looking at the unclassified genera, a positive gradient was recorded from the surface water to the hyporheic sediment where the relative numbers of reads could reach 44.8–55.8%.

**Figure 5:**
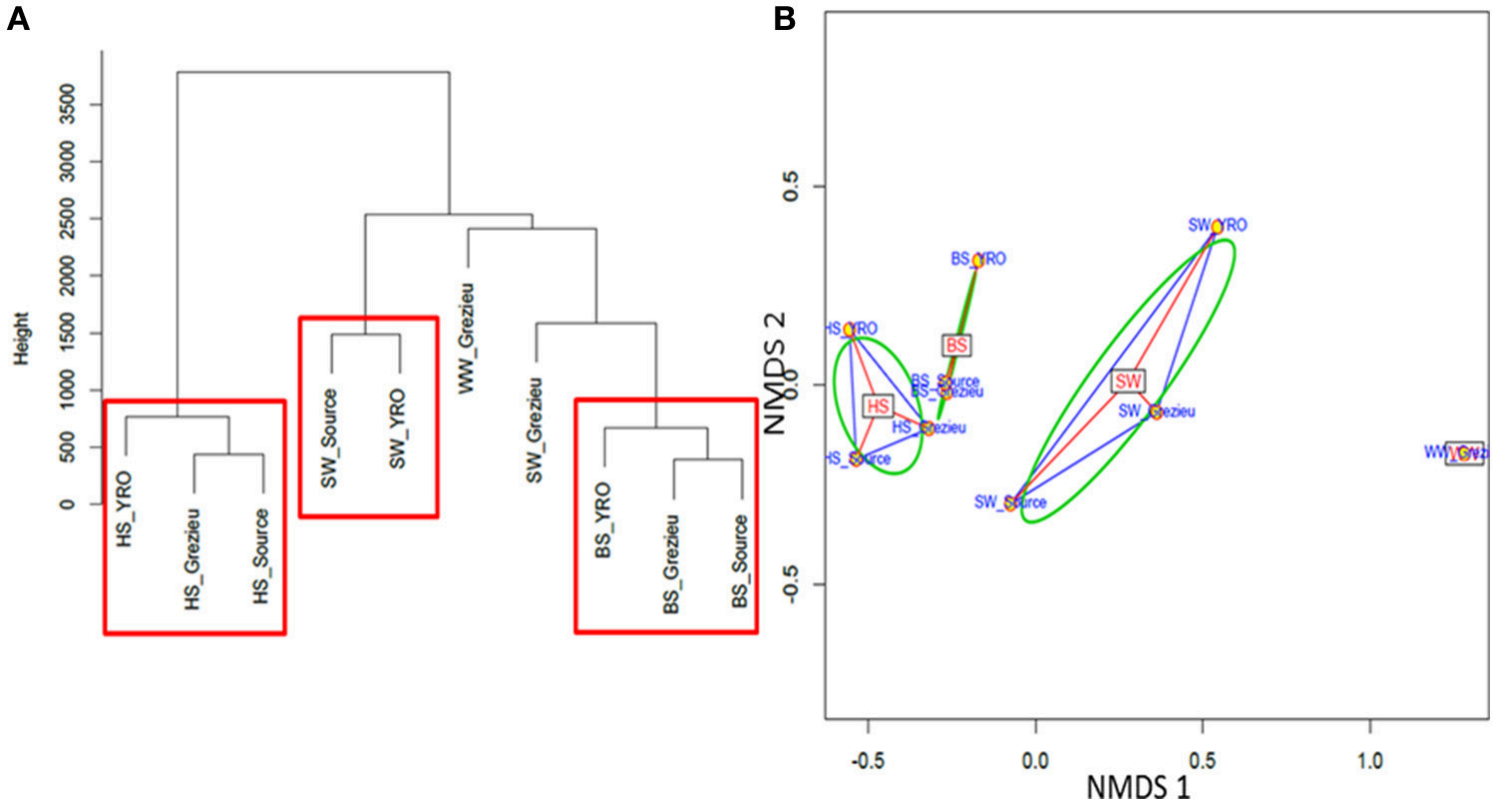
**Ascendant Hierarchical Classification (A)** and Non Metric Multi-Dimensional Scaling **(B)** analyses of 16S rRNA gene OTU. The 50K dataset (5670 reads per sample) containing 16S rRNA gene sequences from surface waters (SW), benthic (BS), and hyporheic sediments (HS), and wastewater (WW), sampled along the Chaudanne river was used (see text). Vertical lines in **(A)** are indicative of the proximity between the OTU distribution patterns.

Ascendant Hierarchic Classification (AHC) and Non Metric Multi-Dimensional Scaling (NMDS) analyses were applied on the OTU contingency table (Figure 5). These analyses confirmed the similarities inferred at the scale of phyla with a segregation of OTUs according to their compartment (WW, SW, BS, or HS) of origin. AHC clearly separated the OTU patterns inferred from the hyporheic sediments from the other ones (Figure 5A). The pattern inferred from the hyporheic zone of the outlet was shown to be distinct but related to those inferred from the source and Grezieu sites. A similar association was observed for the inferred genus patterns of the benthic sediments (Figure 5A). The NMDS analysis was more in line with the inferences made with the phyla dataset (Figure 3). The proximity between the ordinations of the OTU distribution patterns of the hyporheic and benthic sediments was confirmed. In both analyses, AHC and NMDS, the inferred distribution patterns of OTU from wastewaters were found closer to those inferred from surface waters than sediments. The closest similarity between surface water and wastewater OTU patterns was observed for the Grezieu site (Figure 5). Surface water OTU patterns showed a greater heterogeneity than those of the benthic and hyporheic sediments (Figure 5B).

#### MST and NGS pathogen-related *rrs* reads

MST markers used in this study are targeting *rrs* sequences. Their matching sequences were thus searched for in the V5-V6 *rrs* reads. The 50K *rrs* dataset did not harbor these MST related sequences. Nevertheless, they could be recovered from the 100K *rrs* dataset (not considering the surface water dataset from the source). A phylogenetic tree was built to compare the *n* = 683 *Bacteroides* sequences (from 118 OTUs) of the dataset, and the human HF183 and Rum-2-Bac—related sequences (Figure S3). OTU004 was found grouping with the HF183-related sequence. This OTU was made of sequences obtained from wastewater (*n* = 39 sequences), and from the surface waters (*n* = 8) and hyporheic sediments (*n* = 1) of the Grezieu site. This OTU was absent from the other sites. OTU001 had the greatest number of sequences (*n* = 207) and was found related to *Bacteroides* 16S rRNA gene sequences from human fecal material (accession number: AY975390, 100% similarity). OTU001 was recovered from wastewater (*n* = 157), surface waters (*n* = 27) from the Grezieu site and Yzeron river outlet (*n* = 21), and from hyporheic sediments of the Grezieu site (*n* = 1). Rum-2-Bac—related sequences were not detected in the V5-V6 *rrs* reads.

The two datasets (50K and 100K) were further analyzed by the Blast+ tool in order to detect sequences showing high sequence similarity with a local database grouping 16S rRNA gene sequences from waterborne bacterial pathogens (Table S3). Fifteen OTUs among the 50K subset had a 99% or higher identity with these sequences (data not shown). These OTUs showed a total of 595 sequences (data not shown). One of these OTU harbored 469 sequences and was related to a complex of sequences retrieved from *Aeromonas hydrophila* and *Aeromonas caviae* [DNA hybridization groups 1, 2, and 4 according to Carnahan and Joseph (2005)] (100% identity). Most of these sequences (*n* = 396 sequences; 84.4%) came from the wastewater sample but this OTU was also recovered from surface water (*n* = 32), benthic (*n* = 4) and hyporheic sediments (*n* = 2) of the Grezieu site. Samples from the Yzeron outlet also showed this OTU but with a distinct distribution pattern. The hyporheic sediment (*n* = 21) showed more sequences of this OTU than the surface water (*n* = 12) and benthic sediment (*n* = 1). This OTU was detected only in the hyporheic sediment from the source (*n* = 1).

The 100K dataset showed similar trends but more positive hits were recovered (*n* = 28 OTUs for a total of 668 sequences) (Table 2). Globally, 68.7% of these sequences were recovered from wastewater at the Grezieu site (459), and the “source” site was almost pristine (only 2 sequences related to *A. hydrophila/caviae* and *Stenotrophomonas maltophilia*). *A. hydrophila*/*caviae* sequences were the most prevalent. The wastewater sample harbored Otu00014 of this *A. hydrophila*/*A. caviae* complex at high numbers (*n* = 366). The Grezieu site surface waters showed significant but lower numbers (*n* = 56) of this OTU. This OTU was not detected in sediments of the Grezieu site but was recorded among the hyporheic sediment (*n* = 46) and surface water (*n* = 17) at the Yzeron outlet. This OTU was not detected at the source, suggesting a relation between the *A. hydrophila*/*A. caviae* complex and human activities. Some reads were also found related to *rrs* sequences of *S. maltophilia, Acinetobacter baumanii, Pseudomonas aeruginosa, Shigella* sp., and *Nocardia globerula* (Table 2).

**Table 2:** **16S rDNA OTU showing close proximity with reference sequences from pathogenic bacteria, and their distribution among river compartments, recovered from the 100K dataset containing 11040 re-sampled sequences per sample**.

**OTU identity**	**Closest relative**	**% Similarity**	**Number of sequences**	**Distribution pattern between sampling sites for each OTU**									
				**Source**			**Grezieu**				**YRO**		
				SW^*^	**BS**	**HS**	**WW**	**SW**	**BS**	**HS**	**SW**	**BS**	**HS**
Otu00182	*Acinetobacter baumanii*	99.24	57	0	0	0	55	0	2	0	0	0	0
Otu04445	*Acinetobacter baumanii*	99.62	3	0	0	0	0	0	0	0	3	0	0
Otu08216	*Acinetobacter baumanii*	100	1	0	0	0	0	1	0	0	0	0	0
Otu08840	*Acinetobacter nosocomialis*	99.24	1	0	0	0	1	0	0	0	0	0	0
Otu08946	*Acinetobacter nosocomialis*	99.45	1	0	0	0	1	0	0	0	0	0	0
Otu11243	*Acinetobacter nosocomialis*	99.24	1	0	0	0	1	0	0	0	0	0	0
Otu14021	*Acinetobacter baumanii*	99.49	1	0	0	0	1	0	0	0	0	0	0
Otu00797	*Aeromonas hydrophila/A. caviae*	99.62	15	0	0	0	0	0	15	0	0	0	0
Otu00588	*Aeromonas hydrophila/A. caviae*	99.24	19	0	0	0	0	0	0	0	0	19	0
Otu00014	*Aeromonas hydrophila/A. caviae*	100	485	0	0	0	366	56	0	0	17	0	46
Otu17615	*Aeromonas hydrophila/A. caviae*	99.62	1	0	0	0	0	0	0	0	0	1	0
Otu22804	*Aeromonas hydrophila/A. caviae*	99.62	1	0	0	1	0	0	0	0	0	0	0
Otu21941	*Aeromonas hydrophila/ A. caviae*	99.24	1	0	0	0	0	0	0	0	0	0	1
Otu19738	*Nocardia globerula*	99.24	1	0	0	0	0	0	0	0	1	0	0
Otu14572	*Pseudomonas aeruginosa*	99.24	1	0	0	0	0	1	0	0	0	0	0
Otu00363	*Pseudomonas aeruginosa*	100	29	0	0	0	0	0	0	0	0	0	29
Otu19311	*Pseudomonas aeruginosa*	99.62	1	0	0	0	0	0	0	0	1	0	0
Otu00511	*Shigella* sp.	99.24	22	0	0	0	22	0	0	0	0	0	0
Otu04890	*Stenotrophomonas maltophilia*	100	2	0	0	0	0	2	0	0	0	0	0
Otu05004	*Stenotrophomonas maltophilia*	100	2	0	0	0	0	2	0	0	0	0	0
Otu01015	*Stenotrophomonas maltophilia*	100	11	0	0	0	10	1	0	0	0	0	0
Otu06910	*Stenotrophomonas maltophilia*	100	2	0	0	0	0	0	2	0	0	0	0
Otu12264	*Stenotrophomonas maltophilia*	100	1	0	0	0	0	0	1	0	0	0	0
Otu03372	*Stenotrophomonas maltophilia*	100	3	0	0	0	2	0	0	0	0	1	0
Otu22900	*Stenotrophomonas maltophilia*	100	1	0	0	0	0	0	0	0	0	0	1
Otu03529	*Stenotrophomonas maltophilia*	100	3	0	0	0	0	0	0	0	0	3	0
Otu23337	*Stenotrophomonas maltophilia*	100	1	0	0	0	0	0	0	0	0	1	0
Otu25831	*Stenotrophomonas maltophilia*	99.24	1	0	1	0	0	0	0	0	0	0	0

* *OTU distribution patterns for this compartment were inferred from the 50K database. SW, Surface water; BS, benthic sediment; HS, hyporheic sediment*.

## Discussion

Microbiological contaminants found in river systems can have agricultural, urban and industrial origins, can be very diverse, and be released in high amounts. Resistance and resilience of river systems toward such exogenous micro-organisms are to be investigated in order to better establish their incidence on the ecological state of a water system, and infer their relative health hazards for living organisms including man. Rivers are made of sensitive habitats such as the benthic and hyporheic zones which are essential for a great number of aquatic organisms (Fischer and Pusch, 2001). Furthermore, these biotopes harbor indigenous microbial communities which are playing key roles in several chemical transformation processes such as the degradation of organic matter (Vaque et al., 1992; Naegeli and Uehlinger, 1997; Craft et al., 2002). Dumping of allochtonous micro-organisms could impact the ecological equilibria of these habitats, and change their resistance toward undesirable contaminants including bacterial pathogens (Litchman, 2010; Kinnunen et al., 2016). Among microbiological contaminants coming from combined sewer overflows or runoffs, one can detect well-known pathogens of aquatic organisms such as *Pseudomonas aeruginosa* and the Aeromonads which, among others, can infect eels, fishes and frogs (Hubbard, 1981; Panda et al., 2013; Tamam, 2014; Thomas et al., 2014). Furthermore, *E. coli* can be frequently detected, including sometimes the highly virulent shiga-toxin producers.

In this study, we tested the hypothesis considering that most bacterial contaminants coming from agricultural and urban contexts should not be able to persist among river systems and should only be recovered transiently from surface waters and benthic sediments, and rarely from the hyporheic zone. This inability at colonizing river systems would be driven by unfit growth conditions for these bacteria, competition, significant predation or physical barriers preventing their accumulation. MST and NGS of 16S rRNA genes were used and compared in order to test this hypothesis. Detection of ruminant and pig MST fecal pollution indicators were expected among the river samples because several crops amended with manure were found at close vicinity. Furthermore, cattle were observed trampling in one area of the stream. In fact, in this latter area, significant transfers of the bovine MST marker were detected in the hyporheic zone while the human MST marker was not. Such transfers into the hyporheic zone were not detected at the other sampling sites except for the pig MST marker. However, this pig marker showed a peculiar distribution pattern with a greater prevalence in the hyporheic zone than the other compartments. It was previously shown that the pig MST marker could be PCR amplified from other hosts (i.e., muskrat) (Marti et al., 2011a). Specificity tests of such markers are difficult. They mainly concern emperic testings on environmental DNA. In our river samples, one cannot thus exclude cross-reactions with other bacterial groups colonizing the benthic or hyporheic biotopes. Bacteriomes of these compartements have been rarely investigated, and their main components were unknown, at the time the study was initiated. The high ratios obtained for the pig MST marker over the numbers of total *Bacteroidales* plea in favor of a lack of specificity. These ratios were the highest obtained in this investigation. Nevertheless, the ecology of the *Prevotella* cells targeted by this marker remains poorly documented, and we cannot exclude a better acclimation of these cells to the hyporheic zone. This would be an exception in the observed barrier effect of the sediments against fecal bacteria. Nevertheless, pig-2-bac *Prevotella*-like sequences could not be recovered from the NGS 16S rRNA gene datasets of this study. This further supports the probable lack of specificity of this marker. Still, it is to be noted that several environmental factors can explain variations in the occurrence of MST markers released by their host. Microcosm experiments showed temperature to be one of these, with a fast decay of the MST markers in summer (Kreader, 1995; Seurinck et al., 2005; Ballesté and Blanch, 2010). During our investigation, the average water temperature was 5°C. This represented a favorable temperature for the persistence of these markers. In this context, a reliable and significant MST transfer from the surface water to the hyporheic sediments could only be revealed when intense contacts between the host and the river bed had occurred.

Occurrences of Rum-2-Bac and HF183 V5-V6 *rrs* reads in the NGS datasets were also investigated. As for pig-2-bac, Rum-2-Bac DNA sequences could not be detected. However, HF183 closely related but slightly different sequences could be found. OTU004 sequences were allocated to the same *Bacteroides* phylogenetic branch as the HF183 sequence. Nevertheless, the *rrs* NGS datasets did not show the same sensitivity as the MST detection schemes. qPCR appeared to have a better detection threshold of 10^3^–10^4^ copies of targets per 100 mL of surface water or g of sediment while NGS did not lead to a detection of the targeted sequences for most of the investigated samples. The threshold for detecting a particular sequence in a NGS 16S rRNA gene dataset thus appeared to be much higher. The literature is very limited regarding such comparisons of threshold sensitivity of qPCR and NGS. Still, a recent study showed that reverse transcription qPCR required less PCR cycle to give a upper threshold signal than NGS (Illumina MiSeq), when targeting viral DNA (23 Ct against 32 Ct in average, respectively, and corresponding to a difference of about 3 Log units) (Thorburn et al., 2015).

To complete this assessment of NGS datasets for tracking bacterial contaminants, the relation between the numbers of *E. coli*-like DNA sequences in the NGS dataset and *E. coli* plate count numbers was investigated. Variable concentrations of *E. coli* plate count numbers were observed between sampling sites, and appeared to depend upon the investigated river compartment. Seven *E. coli*-related reads (OTU00939) among the wastewaters NGS dataset were identified while about 7 log CFU per 100 mL were recovered by platings. This further confirmed the poor sensitivity of the *rrs* NGS in tracking the well-established FI. It is to be noted that comparisons of *E. coli* plate count numbers were in agreement with a river bed barrier effect. Highest relative *E. coli* counts expressed over the number of total *Bacteroidales* targets were obtained from surface water samples. These ratios were lower for sediment samples and quite variable. This could be the consequence of variable interactions between *E. coli* cells and suspended matter (Rehmann and Soupir, 2009). However, Kovacic et al. (2011) previously reported sediments to concentrate FI. The Chaudanne river geomorphological successions and water regime might explain these differences.

Even though the NGS datasets appeared less sensitive for a tracking of bacterial fecal contaminants, they gave a more global picture of bacterial community changes driven by agricultural and urban practices over the investigated river system (i.e., source, rearing zone, CSO area, and the watershed outlet). NGS *rrs* analyses showed a differentiation of river compartments according to the structure of their bacterial communities. Furthermore, local practices appeared to have impacted V5-V6 *rrs* diversity indices. In fact, Shannon indices for Grezieu site samples were systematically found to be higher than the ones of the “source” samples. This greater diversity was found matching the presence of overflows, delivering bacterial cells at the Grezieu site. Similar changes were observed downstream a river impaired by a wastewater treatment plant (Marti and Balcázar, 2014). However, the diversity of wastewaters was found lower when compared to the ones of river samples. Nutrient inputs from the CSO at the Grezieu site could thus have had a more significant impact on the observed diversity scores than the emitted bacterial taxa. It has been previously shown that nutrient concentrations (P_tot_, C_org_, N_org_) were higher downstream than upstream a CSO (Namour et al., 2015). Moreover, their concentrations were found to be higher in the hyporheic zone than in the benthic one. This supports the hypothesis of an increase of diversity in the hyporheic zone due to wasterwater C-org inputs. This phenomenon could also explain the longitudinal differences between the Grezieu area and the “source” of the river. Similarly, at the Yzeron outlet, because of the presence of a concrete channel, fewer nutrients were probably transferred into the sediments, likely explaining the lower diversity observed among samples collected at this site. Still, it has been suggested that a decrease in diversity can occur from headwater to a river mouth because of the presence of less soil related bacteria and an increase in freshwater specific taxa (Savio et al., 2015).

Regarding the inferred *rrs* taxonomic classifications made in this study, Proteobacteria, Bacteroidetes, and Actinobacteria were found dominating the river samples. Firmicutes were mainly found in wastewater. This is in line with previous studies (Nemergut et al., 2011; Ligi et al., 2014; Liu et al., 2015). However, a decrease of Bacteroidetes OTU numbers among surface waters collected from the Chaudanne tributary and outlet of the watershed was not observed. Their scores remained quite similar between sites. A change in diversity because of modified inputs from soil and riparian zones along the river should have had occurred according to Savio et al. (2015). AHC and NMDS analyses of OTU distribution patterns showed strong similarities between compartments that appeared independent of their localization. *Flavobacterium* was found the most abundant genus among the river samples. *Bacteroidetes* were more represented in the surface waters, and *Acidobacteria* in the hyporheic zone. Interestingly, dominant genera detected among wastewaters such as *Acinetobacter, Aeromonas, Acidovorax, Isobaculum, Pseudomonas*, and *Atopobacter* were found to increase in number among the surface waters at the Grezieu site and Yzeron outlet. A comparison with the source samples suggested a significant impact of a CSO on their relative abundance. Some of these genera are known to harbor significant pathogens such as *P. aeruginosa, A. caviae*, and *A. baumanii*. These bacteria will require a stronger attention in such river systems, and methodologies are needed to improve their tracking and increase our knowledge of their spatio-temporal dynamics.

## Conclusions

Intermittent rivers in peri-urban backgrounds can be impacted by agricultural practices and urban activities, and often experience ecological quality impairments such as loss of species diversity. Their degradation can be due to significant releasing of fecal bacterial contaminants, chemicals, and organic matter. Here, MST and NGS datasets demonstrated the occurrence of significant bacterial community changes in an intermittent stream that were driven by human activities and reared animals. The bovine and human MST markers appeared highly reliable for delimitation of impacted areas but a lack of specificity was observed for the pig MST marker. Bacterial tracking through *rrs* NGS datasets appeared less sensitive, even though some OTU matching human originated *Bacteroides* sequences could be recovered. Nevertheless, these 16S rRNA gene datasets highlighted a differentiation of bacterial community structures according to the sampled compartments. The community structure inferred from the hyporheic zone at the source of the river was found most similar to the one at the outlet of the watershed than the ones of the benthic sediment from the same site. Significant population displacements of indigenous bacteria by exogenous ones did not occur. Most fit aquatic bacteria appeared to remain dominant on the long run, even though highly significant impairments were observed. Still, the river area impacted by CSO events showed increases in diversity among all of its compartments (surface water, benthic and hyporheic sediments) as computed from the NGS datasets. This suggests that a significant number of the wastewater taxa could accumulate in these systems but not in sufficiently high numbers to outcompete the native taxa. Among these, 16S rRNA gene sequences showing high sequence identity with potentially harmful bacteria e.g., *Acinetobacter baumanii* or *Aeromonas hydrophila/caviae* complexes, were detected.

## Author contributions

RM, Analyse of field datasets, meta-taxogenomic analyses and writing. SR, Sampling, meta-taxogenomic analyses. J-BA, Statistical analyses. CC, Sampling and qPCR analyses. SP, Sampling and study design. LM, Sampling and cultural microbiology analyses. MG, MST qPCR analyses. LS, Study design and analysis of datasets. PB, Study design and hydrological analyses. MC, Study design and analysis of datasets. BC, Study design, coordination, analysis of datasets and writing.

### Conflict of interest statement

The authors declare that the research was conducted in the absence of any commercial or financial relationships that could be construed as a potential conflict of interest.
