# Supplementary material for: Human-Driven Microbiological Contamination of Benthic and Hyporheic Sediments of an Intermittent Peri-Urban River Assessed from MST and 16S rRNA Genetic Structure Analyses
Source: Front Microbiol. 2017 Jan 24;8:19. doi: 10.3389/fmicb.2017.00019 (PMC5258724; doi:10.3389/fmicb.2017.00019)
Supplement: Supplementary file 1 [file Table1.DOCX]

Table S1. Hydrological indices defining the impact of CSO events at the experimental site of the Chaudanne River.

Period CSO duration mean flow volume CSO/natural flow Flow - downstream flow <0.001 m^3^.s^-1^ rainfall

number days^1^ (min) (m^3^.s^-1^) (m^3^) (ratio) CSO (m^3^.s^-1^) (% period) (mm)

HFS^2^ – 81 19 9.4 0.011 6.1 0.5 0.015 7.1 238

sampling

time

LFS 10 yrs 105 31 28 0.025 40 3.4 0.009 61.9 430

HFS 10 yrs 67 28 52 0.004 13 0.22 0.020 12.6 290

KS test^3^ 0.11 0.31 <0.001 <0.001 0.08 <0.001 0.03 <0.001 0.01

^1^ yearly number of days with CSO events; ^2^ HFS: high flow season - months 12 to 5; LFS: low flow season - months 6 to 11; ^3^ Non-parametric KS (Kolmogorov-Smirnov) tests between the distributions of the same variables in the LFS and HFS seasons over 10 yrs.
