# Supplementary material for: Human-Driven Microbiological Contamination of Benthic and Hyporheic Sediments of an Intermittent Peri-Urban River Assessed from MST and 16S rRNA Genetic Structure Analyses
Source: Front Microbiol. 2017 Jan 24;8:19. doi: 10.3389/fmicb.2017.00019 (PMC5258724; doi:10.3389/fmicb.2017.00019)
Supplement: Supplementary file 2 [file Table2.DOCX]

Table S2. Physico-chemical parameters of the Chaudanne River samples

| Sample type | Sampling site | Temperature (°C) | pH | Conductance (µS/m) | Relative moisture  (%)^a^ |
| --- | --- | --- | --- | --- | --- |
| *Surface* | 1 | 3.2 | 7.2 | 0.324 | - |
| *water* | 2 | 3.7 | 7.2 | 0.317 | - |
| *(SW)* | 3 | 4.0 | 7.4 | 0.312 | - |
|  | 4 | 4.1 | 7.4 | 0.314 | - |
|  | 5 | 4.6 | 7.4 | 0.312 | - |
|  | 6 | 6.1 | 7.4 | 0.295 | - |
|  | 7 | 7.0 | 7.4 | 0.298 | - |
|  | 8 | 5.4 | 7.4 | 0.313 | - |
|  | 8' | 5.7 | 6.9 | 0.241 | - |
|  | 9 | 6.3 | 7.5 | 0.311 | - |
|  | 10 | 6.4 | 7.6 | 0.257 | - |
|  | 10' | 5.8 | 6.8 | 0.265 | - |
|  |  |  |  |  |  |
| *Benthic* | 1 | 3.4 | 6.8 | 0.280 | 28.7 |
| *sediment* | 2 | - | - | - | 29.5 |
| *(BS)* | 3 | 3.9 | 7.4 | 0.253 | 22.1 |
|  | 4 | 4.1 | 7.4 | 0.299 | 19.3 |
|  | 5 | 4.6 | 7.3 | 0.307 | 23.6 |
|  | 6 | - | - | - | 31.5 |
|  | 7 | 7.0 | 7.3 | 0.289 | 26.7 |
|  | 8 | 5.5 | 7.4 | 0.310 | 25.9 |
|  | 9 | 6.1 | 7.1 | 0.203 | 26.4 |
|  | 10 | 5.7 | 6.7 | 0.235 | 28.2 |
|  | 10' | 5.8 | 6.7 | 0.184 | 23.9 |
|  |  |  |  |  |  |
| *Hyporheic* | 1 | 4.2 | 6.8 | 0.565 | 28.6 |
| *sediment* | 2 | 4.3 | 7.0 | 0.370 | 36.2 |
| *(HS)* | 3 | 4.4 | 7.3 | 0.310 | 26.3 |
|  | 4 | 4.5 | 7.4 | 0.316 | 25.6 |
|  | 5 | 4.8 | 7.5 | 0.305 | 33.1 |
|  | 6 | 5.5 | 7.4 | 0.311 | 26.4 |
|  | 7 | - | - | - | 31.9 |
|  | 8 | 5.7 | 7.4 | 0.295 | 26.9 |
|  | 9 | 6.1 | 6.5 | 0.601 | 27.2 |
|  | 10 | 6.1 | 6.8 | 0.236 | 25.1 |

^a^ %water per g
