# Supplementary material for: Human-Driven Microbiological Contamination of Benthic and Hyporheic Sediments of an Intermittent Peri-Urban River Assessed from MST and 16S rRNA Genetic Structure Analyses
Source: Front Microbiol. 2017 Jan 24;8:19. doi: 10.3389/fmicb.2017.00019 (PMC5258724; doi:10.3389/fmicb.2017.00019)
Supplement: Supplementary file 3 [file Table3.DOCX]

Table S3. List of PCR primers and DNA probes used in this study

| **Primers and probe (target)** | **Sequences (5' -> 3')** | **PCR product (bp)** | **annealing (°C)** | **Final concentration (nmol)** | **Reference** |
| --- | --- | --- | --- | --- | --- |
| **AllBac (Most *Bacteroidales*)** | | | | | |
| AllBac296F | GAGAGGAAGGTCCCCCAC | 106 | 60 | 200 | Layton et al. (2006) |
| AllBac467R | CGCTACTTGGCTGGTTCAG |  |  | 200 |  |
| AllBac375Bhqr | (FAM)CCATTGACCAATATTCCTCACTGCTGCT(BHQ-1) |  |  | 100 |  |
|  |  |  |  |  |  |
| **HF183 (Human-specific *Bacteroidales*)** | | | | | |
| HF183f | ATCATGAGTTCACATGTCCG | 83 | 60 | 200 | Seurinck et al. (2005) |
| HF183r | TACCCCGCCTACTATCTAATG |  |  | 200 |  |
|  |  |  |  |  |  |
| **Pig-2-Bac (Pig-specific *Bacteroidales*)** | | | | | |
| Pig-2-Bac41F | GCATGAATTTAGCTTGCTAAATTTGAT | 116 | 60 | 300 | Mieszkin et al. (2009) |
| Pig-2-Bac163Rm | ACCTCATACGGTATTAATCCGC |  |  | 300 |  |
| Pig-2Bac-113MGB | (VIC)TCCACGGGATAGCC(NFQ-MGB) |  |  | 200 |  |
|  |  |  |  |  |  |
| **Rum-2-Bac (Ruminant-specific *Bacteroidales*)** | | | | | |
| BacB2-590F | ACAGCCCGCGATTGATACTGGTAA | 99 | 60 | 200 | Mieszkin et al. (2010) |
| Bac708Rm | CAATCGGAGTTCTTCGTGAT |  |  | 200 |  |
| BacB2-626P | (FAM)ATGAGGTGGATGGAATTCGTGGTGT(BHQ-1) |  |  | 200 |  |
