# Supplementary material for: Human-Driven Microbiological Contamination of Benthic and Hyporheic Sediments of an Intermittent Peri-Urban River Assessed from MST and 16S rRNA Genetic Structure Analyses
Source: Front Microbiol. 2017 Jan 24;8:19. doi: 10.3389/fmicb.2017.00019 (PMC5258724; doi:10.3389/fmicb.2017.00019)
Supplement: Supplementary file 4 [file Table4.DOCX]

Table S4. List of waterborne bacterial pathogens indicating their main environmental reservoir, health significance, and matching 16S rRNA gene accession number

| Pathogenic bacteria* | Accession number | Environmental reservoir | Health significance |
| --- | --- | --- | --- |
| *Acinetobacter baumanii* | U10874 | Aquatic | Possible (Stewart M and PA Rochelle. 2006) |
| *Aeromonas caviae* | LC050172; AB626132 | Aquatic | Possible (Igbinosa et al., 2012) |
| *Aeromonas hydrophila* | DQ539497 | Aquatic | Possible (Elwitigala et al., 2005) |
| *Aeromonas veronii* | X60414 | Aquatic | Possible (Igbinosa et al., 2012) |
| *Burkholderia* cepacia complexe | X80284; X80286; X80287 | Soil, plants and aquatic | Possible (Chiarini et al., 2006) |
| *Campylobacter* spp. | AY005038; AF302011; DQ531934 | Fecal (man, An)^a^ | Possible (WHO,2006) |
| Pathogenic *Escherichia coli* | BA000007; JASV01000004 | Fecal (man, An) | High (WHO, 2006) |
| *Helicobacter* spp | AF292376; AJ249852; AJ249853; AJ249854; AJ249855; AJ249856; AJ249857; AJ249858; AJ249859; AJ249860; AF292377; AF292378; AF292379; AF292380; AF292381; AF297868; AF237612 | Fecal (man, An) | Possible (Brown, 2000) |
| *Legionella* *pneumophila* | HQ287902; AF122885 | Soil and hydric environment (biofilms, warm waters | High (WHO, 2006) |
| *Leptospira interrogans* | X17547; JQ988857 | Urinary tract (man),waters, soil infected by animals | High (WHO, 2006) |
| *Mycobacterium* avium complex | AF306455 | water and soil | High (WHO, 2006) |
| Nocardia spp. | AF302232; AF302231; AF302230; DQ525592; AJ298932 | water and soil | Possible (OTHU) |
| *Pseudomonas aeruginosa* | DQ518597; AF094713; AF094714; AF094715; AF094716; AF094717; AF094718; AF094719; AF094720; DQ641680 | Aquatic and soil (biofilms), plants, fecal material (man, An), skins (man, An) | High (WHO, 2006) |
| *Salmonella* sp. | AE014613; CP007216; CP000026; CP007523 | Fecal (man, An) | High (WHO,2006) |
| *Shigella* sp. | DQ518591; DQ518595; DQ518598; DQ518602; AM231707; DQ536505; DQ536510; DQ646642 | Fecal (man) | High (WHO, 2006) |
| *Staphylococcus aureus* | DQ630752; DQ630753 | Skins (man), fecal (Hu), Aquatic and soil (biofilms | High (WHO, 2006) |
| *Stenotrophomonas maltophilia* | AJ298934; DQ530116; DQ530118; DQ530130; DQ530138; DQ530140; DQ530144; DQ530153 | Soil, plants, Aquatic | Possible (expert opinion) |
| *Vibrio cholerae* non-O1/nonO139, *V. vulnificus, V. parahaemolyticus* | AB041848; AB041849; AB041850; AB041851; AB041852; AB041853; DQ533691; DQ533692; DQ533693 | aquatic (sea and estuaries, fecal (man and An) | High (OMS, 2006 ; Farama et al, 2008) |
| *Yersinia enterocolitica* | AF282218 | Fecal (man, An) | Possible (Arnone et Walling, 2007) |

* list defined according to WHO (2006) and Lagriffoul *et al*. (2009), ^a^ An: Animal
