# Supplementary material for: Human-Driven Microbiological Contamination of Benthic and Hyporheic Sediments of an Intermittent Peri-Urban River Assessed from MST and 16S rRNA Genetic Structure Analyses
Source: Front Microbiol. 2017 Jan 24;8:19. doi: 10.3389/fmicb.2017.00019 (PMC5258724; doi:10.3389/fmicb.2017.00019)
Supplement: Supplementary file 5 [file Table5.DOCX]

Table S5. Concentrations of fecal bacterial indicators and *Bacteroidales* MST markers along the Chaudanne River.

| Sampling site | Compartment | Fecal indicators (log_10_ CFU / 100 mL or g) | | | |  | MST markers (log_10_ copy number / 100mL or g) | | | | | | | |
| --- | --- | --- | --- | --- | --- | --- | --- | --- | --- | --- | --- | --- | --- | --- |
|  |  | *E. coli* | SD | TTC^c^ | SD |  | Total *Bacteroidales* | SD | Human marker | SD | Ruminant marker | SD | Pig marker | SD |
| 1 | SW | 2.8 | 2.0 | 2.8 | 2.2 |  | 6.4 | 5.8 | 5.2 | - | 3.8 | 3.5 | <LOD | - |
| Mix Ag/Ur | BS | 2.3 | 1.6 | 2.6 | 2.0 |  | 6.8 | 6.6 | <LOD | - | <LOD | - | 4.0 | 3.6 |
|  | HS | <LOD^a^ | - | 0.5 | 0.8 |  | 3.9 | 3.6 | <LOD | - | <LOD | - | <LOD | - |
| 2 | SW | 2.3 | 2.2 | 2.4 | 2.4 |  | 6.3 | 5.7 | 3.5 | 3.3 | 3.7 | 3.6 | <LOD | - |
| Mix Ag/Ur | BS | 1.0 | 1.0 | 1.7 | 1.1 |  | 6.1 | 5.5 | <LOD | - | <LOD | - | 4.0 | 3.5 |
|  | HS | 2.8 | 1.5 | 2.9 | 1.6 |  | 6.8 | 6.2 | <LOD | - | <LOD | - | <LOD | - |
| 3 | SW | 2.1 | 2.1 | 2.1 | 2.1 |  | 6.3 | 6.0 | 3.6 | 3.2 | 3.5 | 2.9 | <LOD | - |
| Mix Ag/Ur | BS | 0.8 | 0.7 | 1.6 | 1.0 |  | 5.6 | 5.4 | <LOD | - | <LOD | - | <LOD | - |
|  | HS | 0.8 | 1.1 | 1.7 | 1.6 |  | 4.8 | 3.9 | <LOD | - | <LOD | - | <LOD | - |
| 4 | SW | 2.4 | 2.1 | 2.5 | 2.2 |  | 6.5 | 6.2 | 3.5 | 3.3 | 3.7 | 3.5 | <LOD | - |
| Mix Ag/Ur | BS | 0.8 | 1.0 | 1.9 | 1.6 |  | 6.0 | 5.4 | <LOD | - | <LOD | - | <LOD | - |
|  | HS | 0.5 | 0.8 | 0.8 | 0.8 |  | 4.9 | 4.4 | <LOD | - | <LOD | - | <LOD | - |
| 5 | SW | 2.8 | 2.2 | 2.9 | 2.3 |  | 7.0 | 6.5 | 3.8 | 3.8 | 3.7 | 3.3 | <LOD | - |
| Mix Ag/Ur | BS | 1.3 | 1.0 | 1.7 | 1.2 |  | 6.0 | 5.6 | <LOD | - | 4.8 | 4.4 | <LOD | - |
|  | HS | 0.5 | 0.8 | 0.8 | 0.8 |  | 5.4 | 4.9 | <LOD | - | <LOD | - | <LOD | - |
| 6 | SW | <LOD | - | <LOD | - |  | 6.3 | 5.9 | 4.7 | 4.0 | 4.3 | 3.9 | <LOD | - |
| Ag | BS | 1.5 | 1.6 | 2.2 | 1.6 |  | 6.7 | 5.9 | <LOD | - | 5.4 | 5.2 | <LOD | - |
|  | HS | 0.8 | 1.1 | 0.8 | 1.1 |  | 4.6 | 4.0 | <LOD | - | <LOD | - | 4.0 | 3.6 |
| 7 | SW | 1.5 | 1.8 | 1.5 | 1.8 |  | 6.6 | 6.1 | <LOD | - | 4.7 | 4.4 | <LOD | - |
| Ag | BS | <LOD | - | 0.8 | 0.8 |  | 6.0 | 5.6 | <LOD | - | 4.8 | 4.5 | <LOD | - |
|  | HS | <LOD | - | <LOD | - |  | 3.7 | 3.4 | <LOD | - | <LOD | - | 3.1 | 2.0 |
| 8 | SW | 2.0 | 2.2 | 2.0 | 2.2 |  | 6.7 | 5.9 | <LOD | - | 5.4 | 5.0 | <LOD | - |
| Ag | BS | 0.8 | 0.8 | 1.4 | 0.8 |  | 6.1 | 5.0 | <LOD | - | 5.1 | 5.0 | <LOD | - |
|  | HS | 0.5 | 0.8 | 1.2 | 1.2 |  | 5.1 | 4.7 | <LOD | - | <LOD | - | 3.6 | 3.3 |
| 8' | SW | 2.5 | 2.2 | 2.6 | 2.3 |  | 7.2 | 6.5 | <LOD | - | 5.8 | 5.1 | <LOD | - |
| Ag | BS | <LOD | - | <LOD | - |  | <LOD | - | <LOD | - | <LOD | - | <LOD | - |
|  | HS | NA^b^ | NA | NA | NA |  | <LOD | - | <LOD | - | <LOD | - | <LOD | - |
| 9 | SW | 2.3 | 2.3 | 2.3 | 2.3 |  | 4.5 | 4.1 | <LOD | - | 3.6 | 3.1 | <LOD | - |
| Ag | SB | 4.4 | 3.5 | 4.5 | 3.5 |  | 8.1 | 7.5 | <LOD | - | 6.5 | 6.0 | <LOD | - |
|  | SH | <LOD | - | <LOD | - |  | 5.4 | 4.9 | <LOD | - | 4.6 | 4.1 | <LOD | - |
| 10 | SW | 1.5 | 1.8 | 1.5 | 1.8 |  | 5.2 | 4.5 | <LOD | - | 3.4 | 2.7 | <LOD | - |
| (Source) | BS | 1.4 | 1.2 | 1.6 | 1.2 |  | 6.6 | 6.2 | <LOD | - | 4.9 | 4.6 | <LOD | - |
|  | HS | 1.1 | 1.1 | 1.1 | 1.1 |  | 4.0 | 3.9 | <LOD | - | <LOD | - | <LOD | - |
| 10' | SW | <LOD | - | 1.5 | 1.8 |  | 5.2 | - | <LOD | - | <LOD | - | <LOD | - |
| Ag | BS | 0.8 | 0.7 | 1.5 | 1.0 |  | 6.3 | 5.6 | <LOD | - | <LOD | - | <LOD | - |
|  | HS | NA | NA | NA | NA |  | <LOD | - | <LOD | - | <LOD | - | <LOD | - |

SW: Surface water; BS: benthic sediment; HS: hyporheic sediment. ^a^: LOD: limit of detection; ^b^: NA: not analyzed, ^c^ TTC: total thermotolorent coliforms. Mix Ag/Ur indicates mix agriculture/urban background, Ag indicates agriculture background
