# Supplementary material for: Human-Driven Microbiological Contamination of Benthic and Hyporheic Sediments of an Intermittent Peri-Urban River Assessed from MST and 16S rRNA Genetic Structure Analyses
Source: Front Microbiol. 2017 Jan 24;8:19. doi: 10.3389/fmicb.2017.00019 (PMC5258724; doi:10.3389/fmicb.2017.00019)
Supplement: Supplementary file 6 [file Table6.DOCX]

| Table S6. Distribution patterns of MST markers between river compartments. | | | | | | | | | | | | | |
| --- | --- | --- | --- | --- | --- | --- | --- | --- | --- | --- | --- | --- | --- |
|  |  |  |  |  |  |  |  |  |  |  |  |  |  |
| MST markers | SW^*^ | | |  | BS^*^ | | |  | HS^*^ | | |  | Fisher's exact test p value |
|  | + | - | %+ |  | + | - | %+ |  | + | - | %+ |  |  |
| Human | 6 | 6 | 50 |  | 0 | 12 | 0 |  | 0 | 12 | 0 |  | <0.014 |
| Ruminant | 10 | 2 | 83 |  | 4 | 8 | 33 |  | 1 | 11 | 8 |  | <0.004 |
| Pig | 0 | 12 | 0 |  | 2 | 10 | 17 |  | 4 | 8 | 33 |  | >0.090 |

^*^ SW: Surface Water, BS: Benthic Sediment, HS: Hyporheic Sediment
