# Supplementary material for: Human-Driven Microbiological Contamination of Benthic and Hyporheic Sediments of an Intermittent Peri-Urban River Assessed from MST and 16S rRNA Genetic Structure Analyses
Source: Front Microbiol. 2017 Jan 24;8:19. doi: 10.3389/fmicb.2017.00019 (PMC5258724; doi:10.3389/fmicb.2017.00019)
Supplement: Supplementary file 7 [file Table7.DOCX]

| Table S7. Bray-Curtis, Chao 1 and Shannon diversity indices on re-sampled 16S rDNA OTU datasets (5670 reads per sample)*.   \|  \| Source \| \| \|  \| Grezieu \| \| \| \|  \| YRO \| \| \| Chao1 [95% confidence interval] \| Shannon [95% confidence interval] \| \| --- \| --- \| --- \| --- \| --- \| --- \| --- \| --- \| --- \| --- \| --- \| --- \| --- \| --- \| --- \| \|  \| SW \| BS \| HS \|  \| WW \| SW \| BS \| HS \|  \| SW \| BS \| HS \| \| SW_Source \|  \|  \|  \|  \|  \|  \|  \|  \|  \|  \|  \|  \| 3244.1 [2922.9-3632.8] \| 5.3 [5.2-5.4] \| \| BS_Source \| 0.752 \|  \|  \|  \|  \|  \|  \|  \|  \|  \|  \|  \| 5581.1 [5116.8-6120.6] \| 6.7 [6.7-6.8] \| \| HS_Source \| 0.901 \| 0.777 \|  \|  \|  \|  \|  \|  \|  \|  \|  \|  \| 6431.5 [5921.6-7018.5] \| 7.2 [7.1-7.2] \| \| WW_Grezieu \| 0.927 \| 0.975 \| 0.987 \|  \|  \|  \|  \|  \|  \|  \|  \|  \| 2632.5 [2239.5-3140.4] \| 4.7 [4.6-4.7] \| \| SW_Grezieu \| 0.849 \| 0.899 \| 0.965 \|  \| 0.875 \|  \|  \|  \|  \|  \|  \|  \| 7065.5 [6058.8-8298.4] \| 5.6 [5.6-5.7] \| \| BS_Grezieu \| 0.796 \| 0.705 \| 0.831 \|  \| 0.946 \| 0.869 \|  \|  \|  \|  \|  \|  \| 9279.3 [8414.1-10275.7] \| 7.1 [7.0-7.1] \| \| HS_Grezieu \| 0.883 \| 0.817 \| 0.777 \|  \| 0.971 \| 0.924 \| 0.734 \|  \|  \|  \|  \|  \| 10071.8 [9202.3-11064.6] \| 7.5 [7.4-7.5] \| \| SW_YRO \| 0.869 \| 0.911 \| 0.959 \|  \| 0.943 \| 0.784 \| 0.905 \| 0.938 \|  \|  \|  \|  \| 1553.4 [1307.4-1885.9] \| 3.5 [3.5-3.6] \| \| BS_YRO \| 0.879 \| 0.736 \| 0.872 \|  \| 0.964 \| 0.939 \| 0.744 \| 0.859 \|  \| 0.902 \|  \|  \| 3208.1 [2892.9-3590.9] \| 5.9 [5.8-5.9] \| \| HS_YRO \| 0.902 \| 0.864 \| 0.859 \|  \| 0.972 \| 0.942 \| 0.861 \| 0.859 \|  \| 0.957 \| 0.898 \|  \| 4893.5 [4514.4-5334.6] \| 6.8 [6.8-6.9] \| |
| --- | --- | --- | --- | --- | --- | --- | --- | --- | --- | --- | --- | --- | --- | --- | --- | --- | --- | --- | --- | --- | --- | --- | --- | --- | --- | --- | --- | --- | --- | --- | --- | --- | --- | --- | --- | --- | --- | --- | --- | --- | --- | --- | --- | --- | --- | --- | --- | --- | --- | --- | --- | --- | --- | --- | --- | --- | --- | --- | --- | --- | --- | --- | --- | --- | --- | --- | --- | --- | --- | --- | --- | --- | --- | --- | --- | --- | --- | --- | --- | --- | --- | --- | --- | --- | --- | --- | --- | --- | --- | --- | --- | --- | --- | --- | --- | --- | --- | --- | --- | --- | --- | --- | --- | --- | --- | --- | --- | --- | --- | --- | --- | --- | --- | --- | --- | --- | --- | --- | --- | --- | --- | --- | --- | --- | --- | --- | --- | --- | --- | --- | --- | --- | --- | --- | --- | --- | --- | --- | --- | --- | --- | --- | --- | --- | --- | --- | --- | --- | --- | --- | --- | --- | --- | --- | --- | --- | --- | --- | --- | --- | --- | --- | --- | --- | --- | --- | --- | --- | --- | --- | --- | --- | --- | --- | --- | --- | --- | --- |

^*^ SW: Surface Water, BS: Benthic Sediment, HS: Hyporheic Sediment
