# Supplementary figures and images for: Human-Driven Microbiological Contamination of Benthic and Hyporheic Sediments of an Intermittent Peri-Urban River Assessed from MST and 16S rRNA Genetic Structure Analyses
Source: Front Microbiol. 2017 Jan 24;8:19. doi: 10.3389/fmicb.2017.00019 (PMC5258724; doi:10.3389/fmicb.2017.00019)

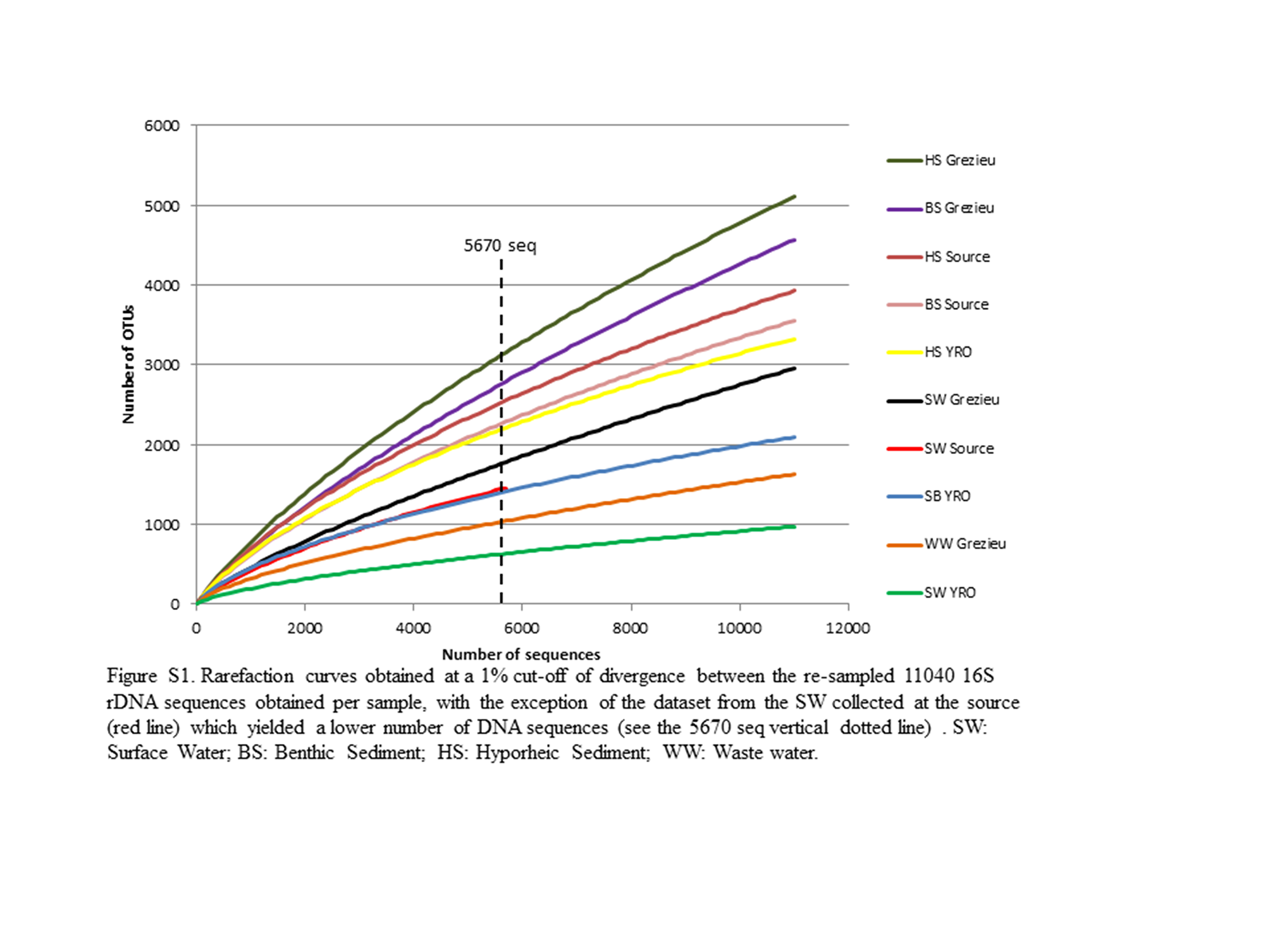

Supplement: Supplementary file 8 [file Image1.TIF]

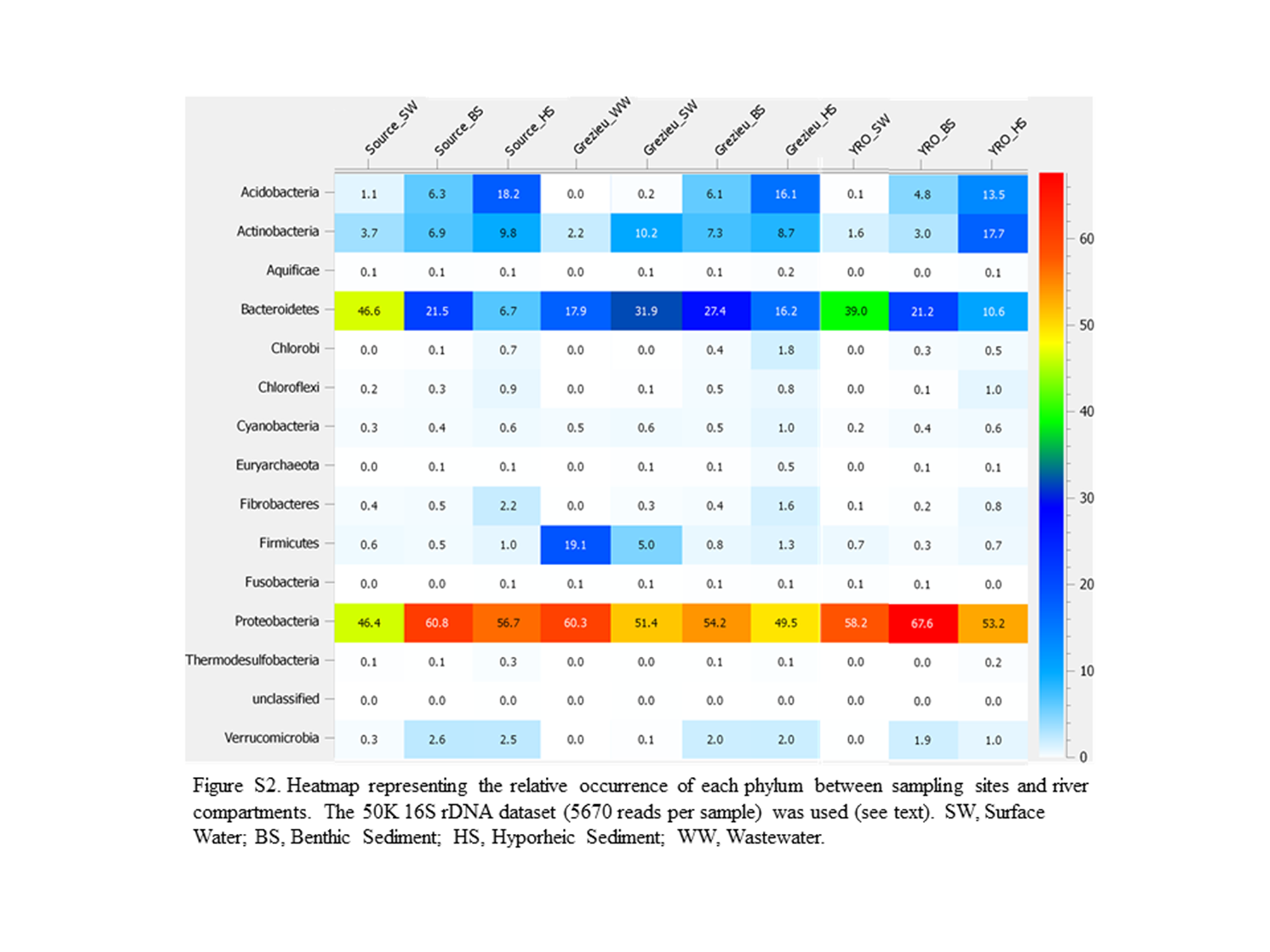

Supplement: Supplementary file 9 [file Image2.TIF]

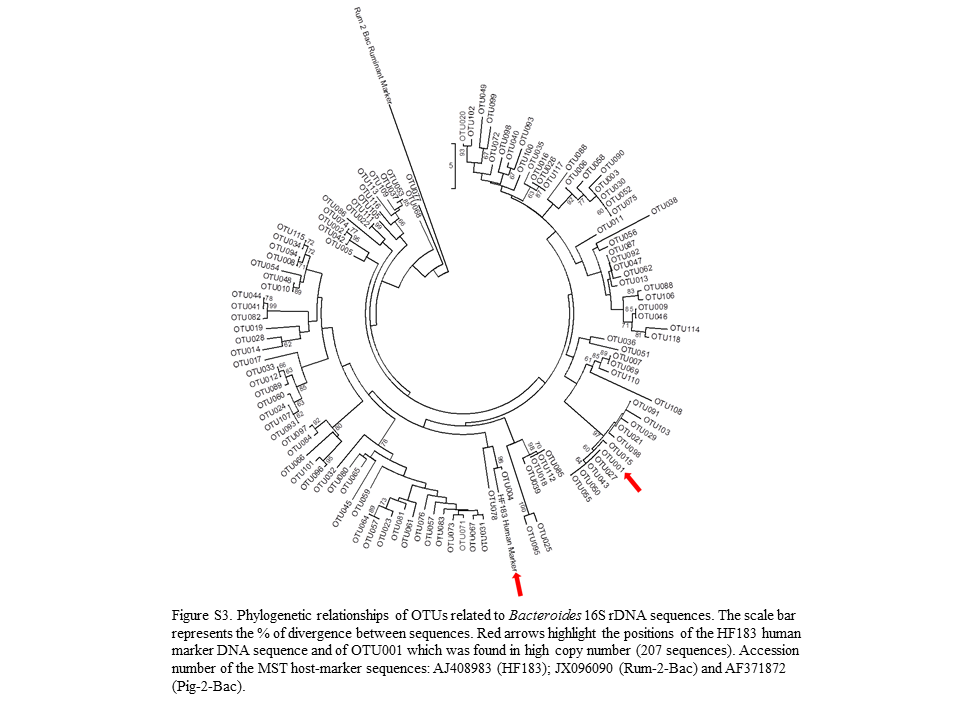

Supplement: Supplementary file 10 [file Image3.TIF]
